# Observation of the single-ion magnet behavior of d^8^ ions on two-coordinate Co(i)–NHC complexes†
†Electronic supplementary information (ESI) available. CCDC 1058164–1058166. For ESI and crystallographic data in CIF or other electronic format see DOI: 10.1039/c5sc02611c


**DOI:** 10.1039/c5sc02611c

**Published:** 2015-09-10

**Authors:** Yin-Shan Meng, Zhenbo Mo, Bing-Wu Wang, Yi-Quan Zhang, Liang Deng, Song Gao

**Affiliations:** a Beijing National Laboratory of Molecular Science , State Key Laboratory of Rare Earth Materials Chemistry and Applications , College of Chemistry and Molecular Engineering , Peking University , Beijing 100871 , P. R. China . Email: gaosong@pku.edu.cn; b State Key Laboratory of Organometallic Chemistry , Shanghai Institute of Organic Chemistry , Chinese Academy of Sciences , 345 Lingling Road , Shanghai 200032 , P. R. China . Email: deng@sioc.ac.cn; c Jiangsu Key Laboratory for NSLSCS , School of Physical Science and Technology , Nanjing Normal University , Nanjing 210023 , P. R. China . Email: zhangyiquan@njnu.edu.cn

## Abstract

The slow magnetic relaxation typical for single-ion magnets has been known for certain low-coordinate 3d metal complexes with d^6^, d^7^, and d^9^ electronic configurations, but never for d^8^ complexes. Herein, we report a study on two-coordinate d^8^ cobalt(i)–N-heterocyclic carbene complexes, for which slow magnetic relaxation behavior was observed for [Co(IMes)_2_][BPh_4_] (IMes: 1,3-dimesitylimidazol-2-ylidene) under an applied dc field. The system represents the first d^8^ single-ion magnet, and features a fitted energy barrier of *U*_eff_ = 21.3 cm^–1^ and pre-exponential factor of *τ*_0_ = 6.6 × 10^–6^ s. The analog two-coordinate cobalt(i) complexes with different NHC ligands, [Co(sIMes)_2_][BPh_4_] (sIMes: 1,3-dimesitylimidazolin-2-ylidene) and [Co(IAd)_2_][BAr^F^_4_] (IAd: 1,3-dimesitylimidazol-2-ylidene; BAr^F^_4_: tetra(3,5-ditrifluoromethylphenyl)borate), do not show such single-ion magnet behaviour. *Ab initio* calculations imply that the dihedral angle between the two NHC planes and the degree of unsaturation of the NHC ligands can dramatically alter the *D* value of the two-coordinate cobalt(i)–NHC ions, possibly *via* changing of the Co–NHC π-interactions, and hence affect the spin–orbit coupling splitting.

## Abstract 2

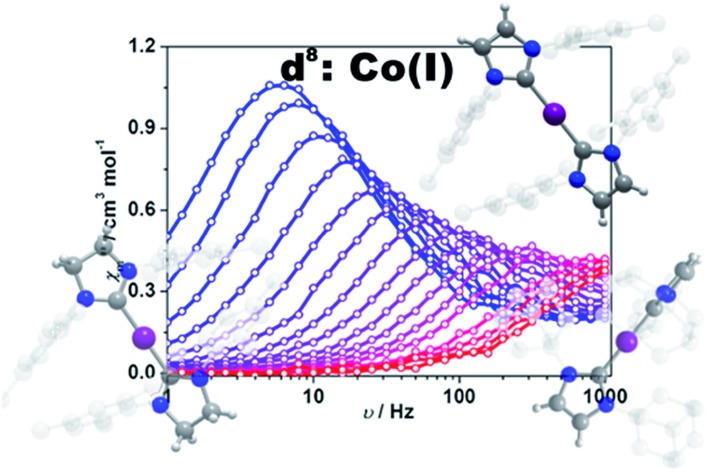
The first single-ion magnet (SIM) [Co(IMes)_2_][BPh_4_] (IMes: 1,3-dimesitylimidazol-2-ylidene) with d^8^ electronic configuration has been found in two-coordinate Co(i)–NHC complexes.

## Introduction

The great interest in low-coordinate 3d metal complexes has been fuelled not only by their inherent synthetic challenge, as this type of species can be prone to disproportionation and coordination with exogenous ligands, but also by their useful reactivity in mediating small molecule activation and catalysis,^1^ and more recently, by their unique magnetic properties that point to the potential of low-coordinate 3d metal complexes as single-ion magnets.^2^ Low-coordinate 3d complexes can feature relatively weak and highly anisotropic ligand-fields, within which a metal ion could exhibit large magnetic moments and even slow magnetization relaxation behavior.1e,^2^,^3^ Notable examples of 3d single-ion magnets have been known for the d^6^ iron(ii) complexes [Fe(C(SiMe_3_)_3_)_2_],^4^ [Fe(N(SiMe_3_)(Dipp))_2_],^4^ [Fe(NHAr*)_2_] (Ar* denotes a bulky aryl group),^4^ [Fe(OAr*)_2_],^4^ and [Fe(N(SiMe_3_)_2_)_2_(PCy_3_)];^5^ the d^7^ iron(i) and cobalt(ii) complexes [K(crypt-222)][Fe(C(SiMe_3_)_3_)_2_],^6^ [Fe(cAAC)_2_Cl] (cAAC denotes cyclic alkylaminocarbene ligands),^7^ [Fe(cAAC)_2_][B(C_6_F_5_)_4_],^7^ [Co(N(SiMe_3_)_2_)_2_L] (L = THF, PCy_3_),5*b* [Li(15-*c*-5)][Co(N(SiMe_3_)_2_)_3_], [dmp_2_Nin(Co(N(SiMe_3_)_2_))_2_]^–^ and [dmp_2_Nin(Co(N(SiMe_3_)_2_(OEt_2_)))_2_]^+^ (dmp_2_Nin = bis(2,6-dimethylphenyl)nindigo radical);5*b* as well as the d^9^ nickel(i) complex [Ni(6-Mes)_2_]Br (6-Mes denotes 1,3-dimesityl-3,4,5,6-tetrahydropyrimidin-2-ylide).^8^ In addition to these, it is expected that nickel(ii) complexes could be very promising candidates for d^8^ single-ion magnets as some nickel(ii) compounds show very large magnetic anisotropy and plenty of low-coordinate nickel(ii) complexes are known.^9^

**Scheme 1:**
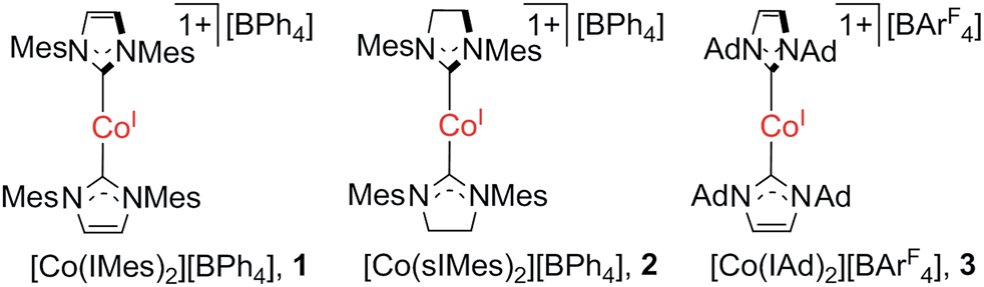
Two-coordinate cobalt(i)–NHC complexes.

However, to our knowledge, no single-ion magnet behavior has been noticed for d^8^ complexes yet.2*c* The status quo warrants further study on new d^8^ complexes, and two-coordinate NHC-transition metal complexes have caught our attention. In this regard, we report herein the synthesis, structure, and magnetic properties of three two-coordinate cobalt(i) complexes with N-heterocyclic carbene (NHC) ligation (**1–3** in Scheme 1). The different NHC ligands in **1–3** have rendered these low-coordinate d^8^ complexes with distinct magnetic properties, among which the slow magnetic relaxation typical for single-ion magnets has been observed for a d^8^ transition-metal complex for the first time. Moreover, theoretical studies disclosed that the dihedral angle between the two NHC planes and the degree of unsaturation of the NHC ligands could dramatically affect the magnetic properties of the low-coordinate cobalt(i)–NHC complexes.

## Experimental

All synthetic experiments were performed under an atmosphere of dry dinitrogen with the rigid exclusion of air and moisture using a standard Schlenk line, or a glovebox. All organic solvents were freshly distilled from sodium benzophenone ketyl immediately prior to use. sIMes,10*a* IAd,10*b* Co(PPh_3_)_3_Cl,10*c* Co(IAd)(PPh_3_)Cl,10*d* and [(IMes)_2_Co][BPh_4_]10*e* were prepared according to reported procedures. All other chemicals were purchased from either Strem or J&K Chemical Co. and were used as received unless otherwise noted. ^1^H NMR spectra were recorded on a VARIAN 400 MHz or Agilent 400 MHz spectrometer. The chemical shifts were reported in units of ppm, referenced to the residual protons of the deuterated solvents for the proton chemical shifts. The elemental analysis was performed by the Analytical Laboratory of the Shanghai Institute of Organic Chemistry (CAS). The absorption spectra were recorded with a Shimadzu UV-3600 UV-vis-NIR spectrophotometer.

### Preparation of Co(sIMes)_2_Cl

To a THF (15 mL) solution of sIMes (0.611 g, 2.0 mmol), Co(PPh_3_)_3_Cl (0.881 g, 1.0 mmol) was slowly added at room temperature, during which time the color of the solution turned from pale yellow to deep reddish brown. After being stirred overnight and removal of the solvent, the residue was washed with *n*-hexane (5 mL × 3) and then extracted with benzene (5 mL). After filtration, the benzene solution was recrystallized by vapor diffusion of *n*-hexane into the solution to afford Co(sIMes)_2_Cl as a red crystalline solid (565 mg, 80%). ^1^H NMR (C_6_D_6_): *δ* 73.64 (2H, C*H*N), 0.61 (6H, *o*-C*H*_3_), –15.49 (3H, *p*-C*H*_3_), –22.44 (2H, C_6_*H*_2_). Anal. calcd for C_42_H_52_ClCoN_4_: C, 71.32; H, 7.42; N, 7.92. Found: C, 71.34; H, 7.47; N, 7.85.

### Preparation of [Co(sIMes)_2_][BPh_4_] (**2**)

To a THF (15 mL) solution of Co(sIMes)_2_Cl (0.703 g, 1.0 mmol), Na[BPh_4_] (0.342 g, 1.0 mmol) was slowly added, during which time the color of the solution turned from deep reddish brown to orange. After being stirred for 12 h and removal of the solvent, the residue was washed with ether (5 mL × 3) and extracted with THF (5 mL). The yellow extraction was filtrated and a small portion of toluene (1 mL) was added. The slow evaporation of THF afforded **2** as an orange crystalline solid (0.691 g, 70%). Absorption spectrum (THF): *λ*_max_(*ε*) = 412 (2580) nm. The ^1^H NMR spectrum of this paramagnetic complex displayed seven characteristic peaks in the range –22.65 to 66.86 ppm. ^1^H NMR (400 MHz, THF-*d*_8_): *δ* 66.86 (8H, C*H*), 10.70 (8H, C_6_*H*_5_), 9.06 (8H, C_6_*H*_5_), 8.41 (4H, C_6_*H*_5_), –12.35 (12H, C*H*_3_), –21.19 (24H, C*H*_3_), –22.65 (8H, C_6_*H*_2_). Anal. calcd for C_66_H_72_BCoN_4_: C, 79.99; H, 7.32; N, 5.65. Found: C, 79.95; H, 7.27; N, 5.52.

### Preparation of [Co(IAd)_2_][BAr^F^_4_] (**3**)

To a THF (15 mL) solution of Co(IAd)(PPh_3_)Cl (0.694 g, 1.0 mmol), IAd (338 mg, 1.0 mmol) was added. After being stirred for 15 min, Na[BAr^F^_4_] (Ar^F^ = 3,5-(CF_3_)_2_–C_6_H_3_, 0.886 g, 1.0 mmol) was added slowly, during which time the color of the solution turned from reddish brown to brown. The mixture was further stirred at room temperature for 12 h. After the removal of the solvent, the residue was washed with *n*-hexane (5 mL × 3) and extracted with THF (5 mL). The yellow green extraction was filtrated and a small portion of toluene (1 mL) was added. The slow evaporation of THF afforded **3** as a green crystalline solid (0.795 g, 50%). Absorption spectrum (THF): *λ*_max_(*ε*) = 315 (1920), 368 (2810) nm. The ^1^H NMR spectrum of this paramagnetic complex displayed seven characteristic peaks in the range –22.65 to 66.86 ppm. ^1^H NMR (400 MHz, THF-*d*_8_): *δ* 50.13, 7.77 (8H), 7.56 (C_6_*H*_5_), 4.69, –6.34, –15.92, –79.52. Anal. calcd for C_78_H_76_BCoF_24_N_4_: C, 58.73; H, 4.80; N, 3.51. Found: C, 58.92; H, 4.79; N, 3.57.

### Magnetic measurements

All the samples were fixed by eicosane and parafilm to avoid movement during the measurements and were sealed in a glass tube to avoid reaction with moisture and oxygen. Direct current susceptibility and alternative current susceptibility measurements with frequencies ranging from 1 to 997 Hz were performed on a Quantum Design MPMS XL-5 SQUID magnetometer for the polycrystalline samples. All the dc susceptibilities were corrected for the diamagnetic contributions from the sample holder, eicosane and from the molecule, using Pascal's constants.^11^

### Computational details

Complete active space second-order perturbation theory (CASPT2), considering the effect of the dynamical electronic correlation based on a complete-active-space self-consistent field (CASSCF) approach, was performed on the cations [Co(IPh)_2_]^1+^, [Co(sIPh)_2_]^1+^, and [Co(IAd)_2_]^1+^ to obtain the parameters *D* and *E* using a MOLCAS 7.8 program package.^12^ The structures of the cations [Co(IPh)_2_]^1+^ and [Co(sIPh)_2_]^1+^ were built based on the structures of the cations in the crystal structures of **1** and **2**, respectively, using hydrogen atoms as replacements for all the methyl groups. The structure of the cation [Co(IAd)_2_]^1+^ in the crystal structure of **3** was directly used for calculation without further modification. The basis sets used for all the atoms were atomic natural orbitals from the MOLCAS ANO-RCC library: ANO-RCC-VTZP for the magnetic center ion Co^I^; VTZ for close C; and VDZ for distant atoms. These calculations employed the second order Douglas–Kroll–Hess Hamiltonian, where scalar relativistic contractions were taken into account in the basis set. After the first CASSCF calculation, the effect of the dynamical electronic correlation was applied using CASPT2. And then, the spin–orbit coupling was handled separately in the restricted active space state interaction (RASSI-SO) procedure. The active electrons in 10 active spaces include eight 3d electrons, and there are 25 mixed spin-free states (from 10 triplets and from 15 singlets). The coordinates of the structures can be found in the ESI† as an xyz file.

## Results and discussion

**Scheme 2:**
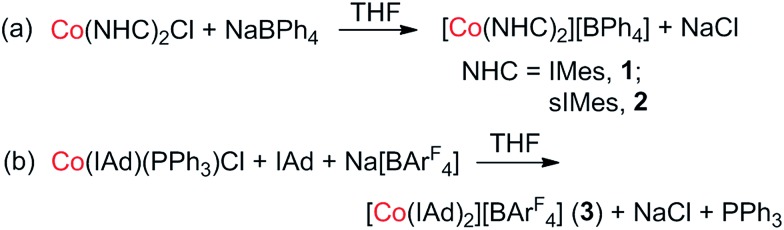
Preparation routes for the two-coordinate cobalt(i) complexes (a) [Co(sIMes)_2_][BPh_4_] and (b) [Co(IAd)_2_][BAr^F^_4_].

Previously, we have reported the synthesis of [Co(IMes)_2_][BPh_4_] (**1**) *via* the salt elimination reaction of Co(IMes)_2_Cl with NaBPh_4_.10*e* The preparation of this first two-coordinate cobalt(i) complex prompted further synthetic efforts towards other two-coordinate cobalt(i) NHC compounds. By applying a similar synthetic route (eqn (a) in Scheme 2), the sIMes complex [Co(sIMes)_2_][BPh_4_] (**2**) was then prepared in a 70% isolated yield as orange crystals. The IAd complex [Co(IAd)_2_][BAr^F^_4_] (**3**), on the other hand, was obtained from the reaction of Co(IAd)(PPh_3_)Cl10*d* with equimolar amounts of Na[BAr^F^_4_] and IAd, in a 50% yield as green crystals (eqn (b) in Scheme 2).^13^

These low-coordinate cobalt(i) complexes are stable at room temperature both in the solid state and in solution under a nitrogen atmosphere. The ^1^H NMR spectrum of **2** measured in THF-*d*_8_ exhibits four broad peaks corresponding to the resonances of the metal-bound NHC ligands at 66.86, –12.35, –21.19, and –22.65 ppm, and that of **3** shows five broad peaks at 50.13, 4.69, –6.34, –15.92, and –79.52 ppm. The peak patterns indicate an idealized *C*_2_ symmetry for the cations in solution and free rotation of the adamantyl groups around the N–C bonds. The absorption spectrum of **2** displays one strong charge-transfer band at 412 nm, which is consistent with the charge-transfer band at 413 nm observed in the spectrum of **1**,10*e* whereas that of **3** appears at 368 nm. In addition to these strong bands, weak absorptions at around 600 nm with an absorption coefficient of *ca.* 200 mol^–1^ L cm^–1^ were noticeable for both complexes (**2** and **3**), which might correspond to the ligand-field transitions of the two-coordinate d^8^ ions.1*e*

**Fig. 1:**
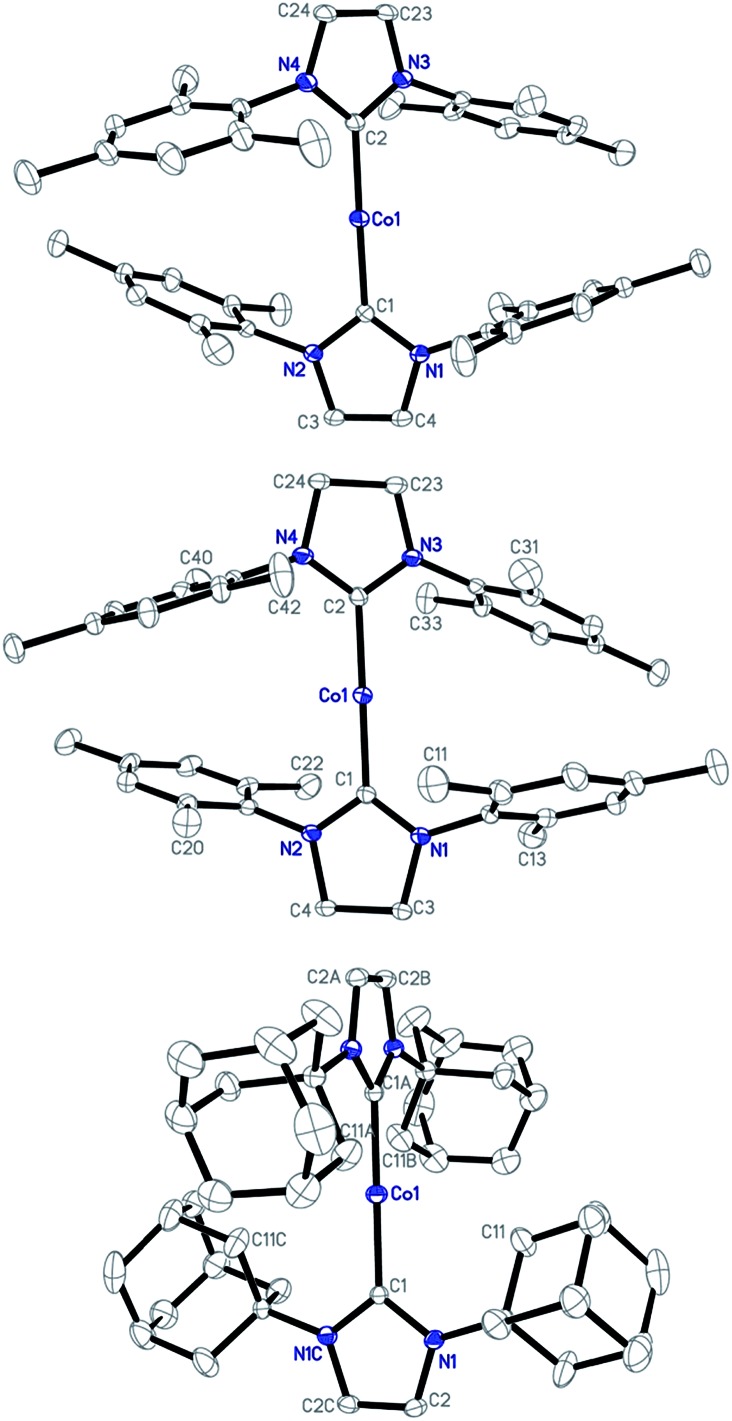
Structures of the cations [Co(IMes)_2_]^+^ of **1** (top), [Co(sIMes)_2_]^+^ of **2** (middle), and [Co(IAd)_2_]^+^ of **3** (bottom) showing 30% probability ellipsoids and the partial atom numbering schemes.

Single-crystal X-ray diffraction studies have established the structures of **1–3** as two-coordinate cobalt(i) complexes (Fig. 1).^11^Table 1 summarizes their key structural parameters. In the structures of **1–3**, even the shortest Co···Co separations are all longer than 9 Å, and no hydrogen-bonding or arene–arene π-interactions are present. Therefore, the intermolecular dipole–dipole interactions, if they exist, could be very small. Similar to **1**,10*e* the C(carbene)–Co–C(carbene) alignments in **2** and **3** are also linear (178.4(1) and 180°, respectively). The Co–C(carbene) distance in **2** (1.936(2) Å) is identical to that of **1**, and is slightly shorter than that of **3** (1.943(3) Å). As compared to their counterpart in the cAAC complex [Co(Et_2_-cAAC)_2_][BAr^F^_4_] (Et_2_-cAAC: 1-(2′,6′-diisopropylphenyl)-3,3-diethyl-5,5-dimethylpyrrolidine-2-ylidene) (1.957(2) Å),^14^ the Co–C(carbene) bonds in **1–3** are shorter. One of the apparent structural differences among **1–3** is the dihedral angle between the five-membered NHC planes. The presence of the IAd ligands in **3**, which are the most sterically demanding among the three NHC ligands,^15^ rendered vertical alignment of the two planes, whereas, smaller dihedral angles of 39.55 and 35.02° are observed for the structures of **1** and **2**, respectively. Another important structural difference is the weak interaction of the cobalt centers with the N-bonded substituents. Apparent secondary metal–NHC interactions between the cobalt center and the adamantyl groups with a shortest Co···C distance of 2.878 Å are evidenced in **3**. As for the structures of **1** and **2**, the shortest Co···C distances involving the *ortho* methyl groups of the flanking mesityls and the cobalt center are 3.838 and 3.651 Å, respectively, approaching the sum of the van der Waals radii of Co with C (3.7 Å).^16^

**Table 1:** Key distances (Å) and angles (°) of the two-coordinate cations in **1–3**, revealed by XRD

	**1** ^*a*^	**2**	**3**
C–Co–C	178.6(1)	178.4(1)	180
Co–C	1.937(2)	1.936(2)	1.943(3)
*α* ^*b*^	39.55	35.02	90
Co···C^*c*^	3.838	3.651	2.878
Co···Co^*d*^	9.312	9.322	13.496

^*a*^Data from ref. 10.

^*b*^Dihedral angle between the two idealized planes of the five-membered rings of the carbene ligands.

^*c*^The shortest Co···C separation of the cobalt center with the carbon atoms on the N-wingtip.

^*d*^The shortest Co–Co separation.

**Fig. 2:**
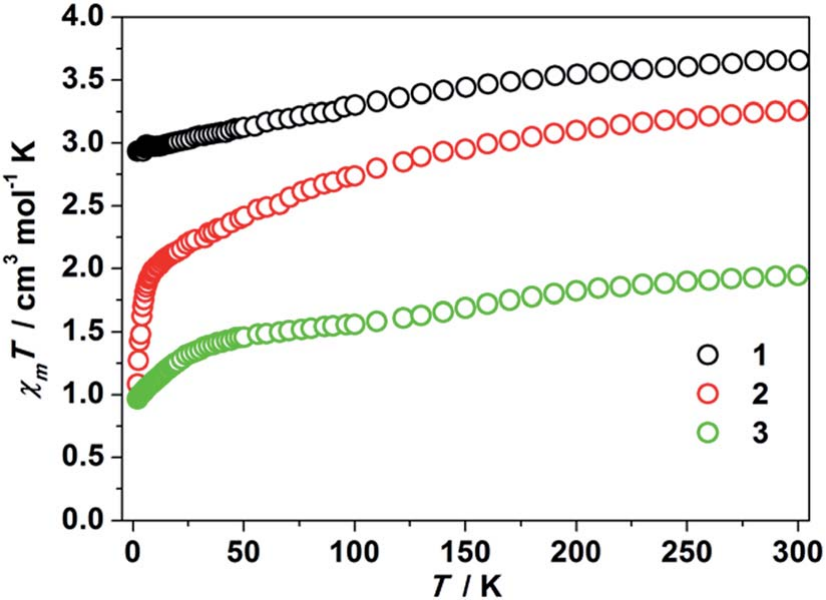
*χ*_m_ *T* products *versus T* plots for **1–3**. These data were collected under a 1 kOe applied dc field.

Some of the two-coordinate iron(i), iron(ii), and nickel(ii) metal complexes featuring high uniaxial symmetry maintain angular momentum, leading to a larger zero field splitting.1c,^4^–^9^ In order to examine whether this is the case for the two-coordinate d^8^ cobalt(i) complexes or not, we performed static magnetic experiments on the solid samples of **1–3**. Magnetic susceptibility measurements confirmed a high magnetic momentum of 3.65 cm^3^ mol^–1^ K (*μ*_eff_ = 5.40 *μ*_B_) and 3.26 cm^3^ mol^–1^ K (*μ*_eff_ = 5.10 *μ*_B_) at room temperature for **1** and **2**, respectively. These values are much higher than the spin-only value of 1 cm^3^ mol^–1^ K for Co(i) of *S* = 1, implying the contribution of unquenched orbital angular momentum. The susceptibility changes of the two complexes at a low temperature range, however, are different. As depicted in Fig. 2, the *χ*_m_*T* value of **1** decreases slowly from 3.65 cm^3^ mol^–1^ K to 2.93 cm^3^ mol^–1^ K when cooling from 300 K to 2 K. For sample **2**, a sharp decrease of the *χ*_m_*T* value from 1.94 cm^3^ mol^–1^ K to 1.08 cm^3^ mol^–1^ K was observed as the temperature changed from 8 K to 2 K. Compared with the previously reported two-coordinate Co(ii) and Co(i) compounds,^17^ the room temperature susceptibility of **1** and **2** is slightly larger, which is probably due to the unquenched angular momentum. Compared to that of **1** and **2**, the magnetic momentum of **3** is much lower (Fig. 2). The *χ*_m_*T* value of **3** at room temperature is 1.94 cm^3^ mol^–1^ K (*μ*_eff_ = 3.94 *μ*_B_). A sharp downturn of its *χ*_m_*T* value occurs below 50 K, and the *χ*_m_*T* value eventually reaches 0.97 cm^3^ mol^–1^ K at 2 K.

The variable-field variable-temperature magnetization experiments (Fig. S5–S7†) confirmed the existence of magnetic anisotropy, which is typical for low coordinate cobalt compounds.1e,17f,h We attempted to use ANISOFIT 2.0 to quantify the zero-field splitting parameters *D* and *E* from the variable-field variable-temperature data of **1–3**. However, no reasonable fits for the effective spin Hamiltonian: *H* = *DS*_z_^2^ + *E*(*S*_*x*_^2^ – *S*_*y*_^2^) + *gμ*_*β*_*SB* were obtained. This is probably due to the remarkable first-order orbital angular momentum contribution of the compounds.^18^ Consequently, complete active space second-order perturbation theory (CASPT2) calculations based on the cations [Co(IPh)_2_]^1+^, [Co(sIPh)_2_]^1+^, and [Co(IAd)_2_]^1+^ were performed to acquire the *D* and *E* values as approximations for those of **1–3**. As shown in Table S1,† the calculated energies of the spin-free states were found to be much larger than the spin–orbit coupling energies, implying that the description using Russell–Saunders coupling is not necessary. On the other hand, the *z* component of the orbital angular moment |*L*_z_| of [Co(IPh)_2_]^1+^ is 0.179, which is much larger than that of the other two complexes, indicating a small orbital contribution (Table S2†). Thus, *m*_s_ can be regarded as a good quantum number for the three complexes, and the zero-field splitting parameters *D* and *E* could be used to depict their magnetic anisotropies. For [Co(IPh)_2_]^1+^, which can be viewed as a simplified model of the cation of **1**, large anisotropic properties are maintained with a positive *D* value of 33.4 cm^–1^ and *E* value of –4.4 cm^–1^, showing strong easy-plane anisotropy. This calculated *D* value is comparable to the early reported nickel(ii) d^8^ complexes.^19^ In contrast, the cation [Co(sIPh)_2_]^1+^, a simplified model for the cation of **2**, possesses a negative *D* value (–8.2 cm^–1^), and the cation [Co(sIAd)_2_]^1+^ of **3** has a negligible value, *D* = –0.11 cm^–1^. These calculated results imply that **1** might possess relatively stronger magnetic anisotropy compared to **2** and **3**, as well as the larger orbital momentum contribution, which is also reflected by their variable-temperature static magnetic susceptibilities.

**Fig. 3:**
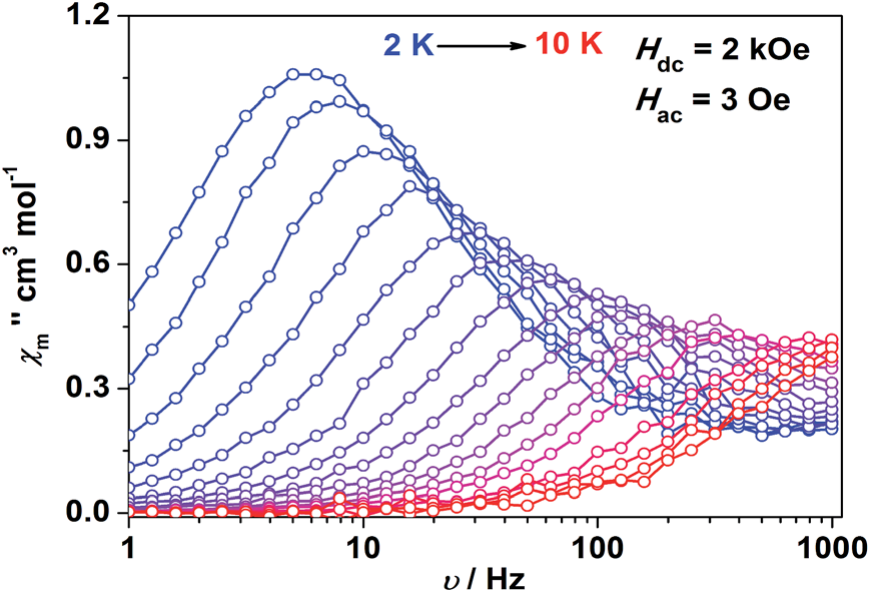
Frequency-dependent out-of-phase component for **1** under a 2 kOe dc field. Solid lines are a guide for the eyes.

**Fig. 4:**
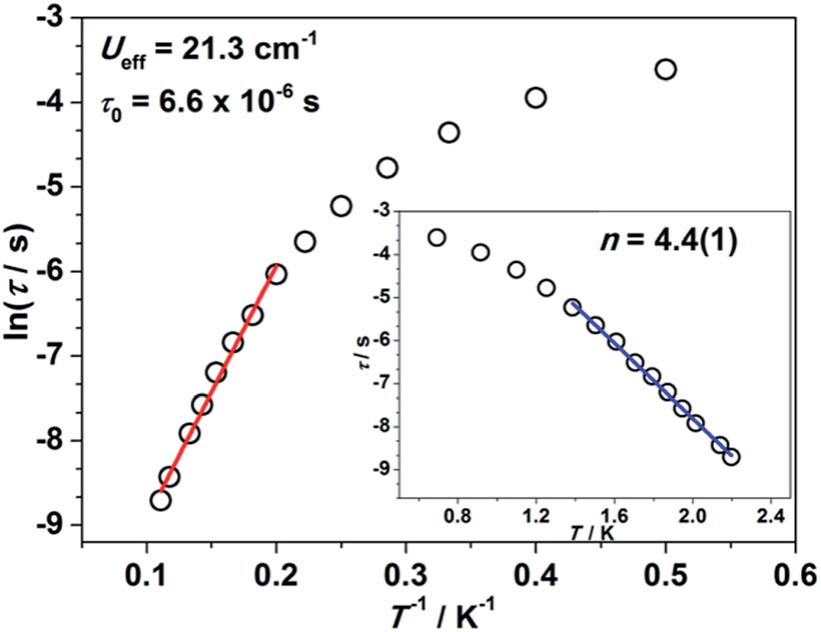
Relaxation time *τ versus* the inverse of temperature *T*^–1^ plot. The red line represents the thermal relaxation fitted by the Arrhenius law. Inset: ln *τ versus* temperature *T* plot. The blue line represents the exponential fitting.

Impressed by the large magnetic anisotropy of **1**, we further performed dynamic magnetic experiments to probe its magnetic relaxation behavior. In the absence of a dc field, a temperature-dependent out-of-phase signal *χ*′′_m_ was observed for **1** while out-of-phase signal peaks were not observed (Fig. S8†). This can probably be attributed to the very fast magnetic tunneling and the relaxation timescale, which is beyond our instruments. This phenomenon has been found in many mononuclear transition metal compounds.^20^ Under an optimized dc field of 2000 Oe, frequency-dependent and out-of-phase peaks were then observed between 2 K and 10 K (Fig. 3 and S9†). The frequency-dependent data can be transformed into Cole–Cole plots (Fig. S10†) and fitted using a generalized Debye model, which gives a fitted distribution of relaxation time, with *α* in the range of 0.04 to 0.2 (Table S3†). Plotting the relaxation time *τ vs. T*^–1^ gives a distinct curve as shown in Fig. 4. Assuming Orbach process character, fitting the relaxation time with the Arrhenius law: *τ* = *τ*_0_ exp(*U*_eff_/*k*_B_*T*) at high temperature gives a linear fit with *U*_eff_ = 21.3 cm^–1^ and *τ*_0_ = 6.6 × 10^–6^ s. The spin-reversal energy barrier falls in the typical range of the reported barriers of cobalt(ii) single-ion magnets, and is also comparable to the reversal barriers of the reported two-coordinate d^7^ iron(i) and d^9^ nickel(i) complexes with carbene ligation. For example, [Fe(cAAC)_2_][B(C_6_F_5_)_4_] has a *U*_eff_ value of less than 20 cm^–1^ under a dc field of 3000 Oe,^7^ and [Ni(6-Mes)_2_]Br features a *U*_eff_ value of 11.8 cm^–1^ under a dc field of 600 Oe.^8^ As compared to the two-coordinate iron(ii) amide complexes,^4^ the *U*_eff_ values are relatively small. We noted that a crossover occurred around 5 K, which means that Orbach relaxation behaviour can not describe the whole process. And the pre-exponential factor *τ*_0_ is larger than the usual value of 10^–8^ s for a typical Orbach process. So other relaxation mechanisms such as Raman processes must also be present. Using the exponential law can give a good fit above 4 K, giving *n* = 4.4(1). These results suggest that an optical acoustic Raman process would be more reasonable. This behavior has also been observed in other mononuclear transition metal single molecule magnets (SMMs) and the still existing curvation might be due to the presence of other processes such as not fully quenched quantum tunneling magnetism (QTM).4a,^21^

As mentioned earlier, some nickel(ii) complexes have shown large magnetic anisotropies,9h–i but so far no precedent d^8^ complex showing slow magnetic relaxation is known. Among all the reported 3d single-ion magnets, the magnetic relaxation usually occurs in Kramers systems or non-Kramers systems with a negative *D* value.^2^ Complex **1** represents the first example of a mononuclear d^8^ complex with a relatively large positive *D* value which shows single-ion magnet behavior. Considering the rough structural similarity of **1** with **2** and **3**, we speculated that the other two complexes would also exhibit magnetic relaxation behavior, as **2** and **3** possess easy-axis anisotropy. However, further investigations revealed that neither of them show such a property even under applied dc fields (Fig. S11–S14†).

The different magnetic properties observed for **1–3** raised the question of the origin of the structural distinctions, which, we think, should lie with the specificity of the NHC–metal interactions. The key structural differences around the two-coordinate metal centers in **1–3** are the dihedral angles between the NHC planes, the degree of unsaturation of the NHC ligands (imidazole-2-ylidene in **1** and **3***versus* imidazolin-2-ylidene in **2**), and the secondary metal–ligand interactions. The difference in the dihedral angles (39.55, 35.02 and 90° for **1–3** respectively) might induce a difference in the Co–C(carbene) π-interactions that could quench orbital angular momentum.^7^ To investigate the influence of the dihedral angle on the magnetic anisotropy, we calculated the *D* and *E* values for a series of structures of [Co(IPh)_2_]^1+^ with the dihedral angle *α* varying from 30° to 90° while keeping the other structural parameters unchanged. As shown in Table 2, the anisotropy parameters show evident dependence on the dihedral angle. When *α* increases from 40° to 50°, the *D* value abruptly decreases from 33.4 cm^–1^ to nearly zero. Upon further increasing the dihedral angle from 50° to 90°, the calculated *D* values do not show a significant change. These results indicate that the large dihedral angle (90°) of **3** would be one of the causes for the quenched spin–orbit coupling that leads to the small magnetic anisotropy.

**Table 2:** Calculated *D* and *E* values (cm^–1^) using a CASPT2 method corresponding to different dihedral angles *α* (°) in [Co(IPh)_2_]^1+^

*α* ^*a*^	30	39.55	50	60	70	80	90
*D*	29.4	33.4	0.04	0.03	–1.2	3.1	–2.02
*E*	2.9	–4.4	–0.008	–0.005	–0.03	–0.2	–0.3

^*a*^Dihedral angle between the two idealized planes of the five-membered rings of the carbene ligands.

The main structural difference of **1** and **2** comes from the degree of unsaturation of the five-membered NHC rings which might induce different d–π interactions and change the “genuine” molecular symmetry. The importance of the molecular symmetry for the magnetism of transition metal and lanthanide SIMs or SMMs is well-documented.^4^,^22^ To further clarify the influence of this factor, we modified the structure of [Co(sIPh)_2_]^1+^ by changing the CH_2_CH_2_ backbone into CHCH, keeping the C–C distance unchanged (see atomic coordinates in Table S11† and the xyz file). The calculated *D* value of this modified structure (denoted as [Co(sIPh′)2]^1+^ with *α* = 35°) was measured as 32.6 cm^–1^, being close to that of [Co(IPh)2]^1+^ with *α* = 40° (33.4 cm^–1^). The change in magnetic anisotropy is understandable as the better π-accepting ability of imidazolin ylidenes *versus* imidazol ylidene^15^ should render more pronounced d–π interactions in [Co(sIPh)_2_]^1+^ and hence results in reduced ligand-field symmetry for [Co(sIPh)_2_]^1+^*versus* [Co(sIPh′)_2_]^1+^. Furthermore, the reduction of symmetry would induce more transverse interactions as more transverse components occurred (see Table S2,† the non-zero value of *L*_*yz*_). These results indicate that the degree of unsaturation of the five-membered NHC rings can also influence the magnetic anisotropy of the two-coordinate cobalt(i)–NHC complexes, which should account for the observed difference of the magnetic properties between **1** and **2**.

In addition to these factors, the difference in the secondary metal–ligand interactions in **1–3** also caught our attention. The ease with which a NHC complex will incur secondary metal–ligand interactions should increase with the increasing bulkiness of the NHC ligands (IMes < sIMes < IAd).^15^ The short contact distances observed in **3** and the cyclometallation reactions of the cobalt–IMes complexes10*e* have indicated the feasibility of these two-coordinate cobalt complexes to form secondary metal–ligand interactions within the molecule. Recently, Neese and co-workers have predicted that secondary metal–ligand interactions could cause vibronic coupling and decrease the magnetic anisotropy and relaxation time of single-molecule magnets.4*b* However, a quantitative account of these metal–ligand interactions needs to include more atoms and orbitals, which is beyond what we can currently handle. Qualitatively, we could infer that, among the three complexes, **1** could be the one incurring the weakest secondary metal–ligand interactions.

## Conclusions

In summary, we have found that the high-spin two-coordinate cobalt(i)–NHC complex [Co(IMes)_2_][BPh_4_] exhibits a large room temperature magnetic moment and slow magnetic relaxation behavior under an applied dc field, which represents the first d^8^ single-ion magnet. In contrast to the IMes complex, the analog two-coordinate cobalt(i) complexes with different NHC ligands, [Co(sIMes)_2_][BPh_4_] and [Co(IAd)_2_][BAr^F^_4_], did not show such single-ion magnet behavior. A comparison of the molecular structures of these three complexes revealed their similar linear C(carbene)–Co–C(carbene) cores, with different dihedral angles between the NHC planes, a different degree of saturation of the NHC ligands and different separation distances of the cobalt center toward its nearest carbon atoms on the N-bonded substituents. *Ab initio* investigations on the two former structural factors show that they can largely alter the value of *D*, which predominates the changing of the Co–C(carbene) π-interactions and the changing of further spin–orbit coupling splitting. The final structural difference reflects the ease with which the cations incur secondary metal–ligand interactions increasing in the order of [Co(IMes)_2_]^+^ < [Co(sIMes)_2_]^+^ < [Co(IAd)_2_]^+^, which may also be an important factor affecting the magnetic properties of these low-coordinate complexes, probably acting in a direct way, different from vibronic coupling.

## Supplementary Material

Supplementary information

Supplementary information

Crystal structure data
