# Supplementary material for: Observation of the single-ion magnet behavior of d8 ions on two-coordinate Co(i)–NHC complexes
Source: Chem Sci. 2015 Sep 10;6(12):7156–62. doi: 10.1039/c5sc02611c (PMC5951210; doi:10.1039/c5sc02611c)
Supplement: Supplementary file 1 [file SC-006-C5SC02611C-s001.pdf]

Electronic Supplementary Material (ESI) for Chemical Science.
This journal is © The Royal Society of Chemistry 2015

# Supporting Information

## *for*

## Observation of the Single-Ion Magnet Behavior of $d^8$ Ions on Two-Coordinate Co(I)-NHC Complexes

Yin-Shan Meng,[a] Zhenbo Mo,[b] Bing-Wu Wang,[a] Yi-Quan Zhang,*,[c] Liang Deng*,[b] and Song Gao*,[a]

[a] *State Key Laboratory of Rare Earth Materials Chemistry and Applications, Peking University, Beijing, P. R. China 100871*

[b] *State Key Laboratory of Organometallic Chemistry, Shanghai Institute of Organic Chemistry, Chinese Academy of Sciences, 345 Lingling Road, Shanghai, P. R. China 200032*

[c] *Jiangsu Key Laboratory for NSLSCS, School of Physical Science and Technology, Nanjing Normal University, Nanjing, 210023, P. R. China.*

zhangyiquan@njnu.edu.cn; deng@sioc.ac.cn; gaosong@pku.edu.cn;

**Table of Contents**

1. Summary of crystal data collection and refinement for $Co(sIMes)_2Cl$, **2** and **3** *page* S3

2. References *page* S4

3. Tables and figures

1) **Table S1.** CASPT2 method calculated spin-free and spin-orbit energies, *D* and *E* values ($cm^{-1}$) of $[Co(IPh)_2]^{1+}$, $[Co(sIPh)_2]^{1+}$ and $[Co(IAd)_2]^{1+}$. *page* S5

2) **Table S2.** Angular moments along the main magnetic axes of the ground state of $[Co(IPh)_2]^{1+}$, $[Co(sIPh)_2]^{1+}$ and $[Co(IAd)_2]^{1}$ on pesudospin S = 1/2. *page* S6

3) **Table S3.** Relaxation fitting parameters from the least-square fitting of the Cole-Cole plots of **1** *page* S7

4) **Tables S3-S9** Atomic Coordinates of Model **C**ompounds in Cartesian Coordinate System. *Page* S8

5) **Figure S1.** Absorption spectra of **2** and **3** *page* S15

6) **Figures S2-S4.** $^{1}H$ NMR spectra of $Co(sIMes)_2Cl$, **2** and **3** *page* S16

7) **Figures S5-S7.** Variable-field isothermal susceptibility data of **1, 2** and **3** *page* S19

8) **Figure S8.** Temperature-dependent ac susceptibility data for **1**. *page* S21

9) **Figure S9.** Frequency dependent in-phase ac susceptibility data for **1** *page* S22

10) **Figure S10.** Cole-Cole plots fit for the determination of the temperature dependence of $\tau$ for **1** under 2 kOe dc field from 2 K to 9 K. *page* S23

11) **Figures S11-12.** Temperature-dependent ac susceptibility data and field-dependent ac susceptibility data at 2 K for **2** *page* S24

12) **Figures S13-14.** Temperature-dependent ac susceptibility data and field-dependent ac susceptibility data at 2 K for **3** *page* S25

## 1. Summary of Data Collection and Refinement for the Crystal Structures

**X-Ray Structure Determinations.** Diffraction-quality crystals were obtained as Co(sIMes)$_2$Cl, **2** and **3**. Crystallizations were performed at room temperature. Crystals were coated with Paratone-N oil and mounted on a Bruker APEX CCD-based diffractometer equipped with an Oxford low-temperature apparatus. Data were collected with scans of 0.3 s/frame for 30 s. Cell parameters were retrieved with SMART software and refined using SAINT software on all reflections. Data integration was performed with SAINT, which corrects for Lorentz polarization and decay. Absorption corrections were applied using SADABS.[4] Space groups were assigned unambiguously by analysis of symmetry and systematic absences determined by XPREP. All structures were solved and refined using SHELXTL.[5] Metal and first coordination sphere atoms were located from direct-methods E-maps; other non-hydrogen atoms were found in alternating difference Fourier synthesis and least-squares refinement cycles and during final cycles were refined anisotropically. Hydrogen atoms were placed in calculated positions employing a riding model. CCDC 1058164-1058166 contain the supplementary crystallographic data for Co(sIMes)$_2$Cl, **2** and **3**. These data can be obtained free of charge from The Cambridge Crystallographic Data Centre via www.ccdc.cam.ac.uk/data_request/cif.

Crystal data for Co(sIMes)$_2$Cl: $C_{42}H_{52}ClCoN_4$, $M_r$ = 707.26, monoclinic, space group $P2(1)/c$, $a$ = 23.591(2), $b$ = 10.4360(11), $c$ = 15.7392(16) Å, $\alpha$ = 90°, $\beta$ = 101.118(2)°, $\gamma$ = 90°, $V$ = 3802.3(7) Å$^3$, $T$ = 140(2) K, $Z$ = 4, $\rho_{calcd}$ = 1.235 g cm$^{-3}$, $2\theta_{max}$ = 64.04°, $\mu(Mo_{K\alpha})$ = 0.71073 Å, absorption corrections applied by using SADABS, relative transmission factors in the range 0.8422-0.9164. A total of 36991 reflections were collected and led to 11550 unique reflections, 11550 of which with $I > 2\sigma(I)$ were considered as observed, $R_1$ = 0.0514, w$R_2$ ($F^2$) = 0.1257.

Crystal data for **2**: $C_{66}H_{72}BCoN_4$, $M_r$ = 991.02, monoclinic, space group $C2/c$, $a$ = 29.581(3), $b$ = 11.9267(11), $c$ = 32.317(3) Å, $\alpha$ = 90°, $\beta$ = 101.110(2)°, $\gamma$ = 90°, $V$ = 11188.2(17) Å$^3$, $T$ = 140(2) K, $Z$ = 8, $\rho_{calcd}$ = 1.177 g cm$^{-3}$, $2\theta_{max}$ = 61.22°, $\mu(Mo_{K\alpha})$ = 0.71073 Å, absorption corrections applied by using SADABS, relative transmission factors in the range 0.6748-0.7461. A total of 54545 reflections were collected and led to 17174 unique reflections, 17174 of which with $I > 2\sigma(I)$ were considered as observed, $R_1$ = 0.0543, w$R_2$ ($F^2$) = 0.1410.

Crystal data for **3**: $C_{78}H_{76}BCoF_{24}N_4$, $M_r$ = 1595.17, triclinic, space group $P4(2)/n$, $a$ = 14.0529(10), $b$ = 14.0529(10), $c$ = 18.2643(18) Å, $\alpha$ = 90°, $\beta$ = 90°, $\gamma$ = 90°, $V$ = 3606.9(5) Å$^3$, $T$ = 140(2) K, $Z$ = 2, $\rho_{calcd}$ = 1.469 g cm$^{-3}$, $2\theta_{max}$ = 61.26°, $\mu(Mo_{K\alpha})$ = 0.71073 Å, absorption corrections applied by using SADABS, relative transmission factors in the range 0.9028-0.9497. A total of

35363 reflections were collected and led to 5535 unique reflections, 5535 of which with $I > 2\sigma(I)$ were considered as observed, $R_1 = 0.0656$, w$R_2$ ($F^2$) = 0.1841.

## 2. References


[1] G. M. Sheldrick, SADABS: Program for Empirical Absorption Correction of Area Detector Data. University of Göttingen: Germany, 1996.

[2] G. M. Sheldrick, SHELXTL 5.10 for Windows NT: Structure Determination Software Programs. Bruker Analytical X-ray systems, Inc.: Madison, Wisconsin, USA, 1997.

**Table S1**. CASPT2 method calculated spin-free and spin-orbit energies, *D* and *E* values ($cm^{-1}$) of $[Co(IPh)_2]^{1+}$, $[Co(sIPh)_2]^{1+}$ and $[Co(IAd)_2]^{1+}$.

| CASPT2 method calculated energies of the low-lying spin-free states | | |
|---|---|---|
| $[Co(IPh)_2]^{1+}$ (α = 39.55°) | $[Co(sIPh)_2]^{1+}$(α = 35°) | $[Co(IAd)_2]^{1+}$ (α = 90°) |
| 0 | 0 | 0 |
| 1076.998 | 1801.005 | 37243.02 |
| 2173.706 | 6526.358 | 40990.33 |
| 20746.71 | 12872.32 | 42368.54 |
| 27522.19 | 21093.18 | 42771.44 |
| 31769.68 | 22500.5 | 50388.62 |
| 32739.38 | 25244.34 | 50717.71 |
| 34453.5 | 26257.79 | 53450.01 |
| 35880.25 | 34501.14 | 54610.42 |
| 39052.78 | 34917.86 | 54937.61 |
| CASPT2 method calculated energies of the low-lying spin-orbit states | | |
| 0. | 0 | 0 |
| 29.0 | 3.10 | 0.041 |
| 37.8 | 12.1 | 0.1 |
| 1047.3 | 1793.0 | 10893.3 |
| 1055.8 | 1800.2 | 12700.1 |
| 1131.2 | 1808.8 | 22930.9 |
| 2212.3 | 6537.8 | 36583.6 |
| 2369.4 | 6579.4 | 37241.8 |
| 2371.0 | 6581.5 | 37242.0 |
| 9107.3 | 9465.8 | 37242.8 |
| 10706.1 | 11251.9 | 40991.1 |
| 12334.8 | 12623.9 | 40992.3 |
| 12404.2 | 12882.1 | 40992.4 |
| 12815.4 | 12884.8 | 42368.6 |
| 20793.2 | 12887.8 | 42368.8 |
| 20811.2 | 19278.8 | 42368.8 |
| Calculated zero-field splitting (ZFS) parameters: the axial *D* and rhombic *E* parameters of the ground manifold (pseudospin S=1) | | |
| $D = 33.4$<br>$E = -4.4$ | $D = -8.2$<br>$E = -3.7$ | $D = -0.07$<br>$E = 0.02$ |

**Table S2**. Angular moments along the main magnetic axes of the ground state of $[Co(IPh)_2]^{1+}$, $[Co(sIPh)_2]^{1+}$ and $[Co(IAd)_2]^{1}$ on pesudospin $S = 1/2$.

| | Angular moments along the main magnetic axes | | | | | | | | |
|---|---|---|---|---|---|---|---|---|---|
| | $[Co(IPh)_2]^{1+}$ | | | $[Co(sIPh)_2]^{1+}$ | | | $[Co(IAd)_2]^{1+}$ | | |
| | $X_m$ | $Y_m$ | $Z_m$ | $X_m$ | $Y_m$ | $Z_m$ | $X_m$ | $Y_m$ | $Z_m$ |
| $L_x$ | 0 | 0 | -0.0163 | 1E-09 | 0 | 0.06596 | 1E-09 | 0 | 0.00014 |
| $L_y$ | -2E-09 | 3E-09 | 0.1552 | 0 | 0 | -0.0205 | 0 | 0 | 0.00146 |
| $L_z$ | 3E-09 | -4E-09 | -0.1792 | -1E-09 | -1E-09 | -0.0820 | 0 | 0 | -0.0008 |
| $S_x$ | 0 | 1E-09 | 0.00814 | -1.2E-08 | -2E-09 | -0.0329 | -3.6E-07 | 0 | -6.8E-05 |
| $S_y$ | 1E-09 | -3E-09 | -0.0775 | -8E-09 | -1.8E-08 | 0.01025 | -3.6E-07 | -7.2E-7 | -0.00073 |
| $S_z$ | 1.5E-08 | -2.1E-08 | -0.9842 | -2.1E-08 | -4E-09 | -0.9978 | 1E-09 | 0 | -1 |

**Table S3**. Relaxation fitting parameters from the least-square fitting of the Cole-Cole plots of **1** according to the generalized Debye model.

| $T$ / K | $\chi_S$ / cm$^3$ mol$^{-1}$ K | $\chi_T$ / cm$^3$ mol$^{-1}$ K | $\alpha$ | $\tau$ / s |
|---|---|---|---|---|
| 2 | 0.8956 | 3.7821 | 0.2051 | 0.02702 |
| 2.5 | 0.7712 | 3.2354 | 0.1453 | 0.01935 |
| 3 | 0.6525 | 2.7642 | 0.1294 | 0.01282 |
| 3.5 | 0.6016 | 2.3756 | 0.0866 | 0.00845 |
| 4 | 0.5439 | 2.0867 | 0.0718 | 0.00536 |
| 4.5 | 0.4675 | 1.8534 | 0.0719 | 0.00353 |
| 5 | 0.4537 | 1.6627 | 0.0695 | 0.00241 |
| 5.5 | 0.3362 | 1.5297 | 0.0711 | 0.00148 |
| 6 | 0.3358 | 1.3949 | 0.0456 | 0.00107 |
| 6.5 | 0.2835 | 1.2867 | 0.0335 | 0.000751 |
| 7 | 0.2638 | 1.1953 | 0.01242 | 0.000515 |
| 7.5 | 0.1764 | 1.1171 | 0.03969 | 0.000365 |
| 8.5 | 0.1385 | 0.9806 | 0.02566 | 0.000218 |
| 9 | 0.0745 | 0.9338 | 0.02154 | 0.000165 |

**Table S4**. Atomic coordinates of model $[Co(IPh)_2]^{1+}$ in Cartesian coordinate system. The dihedral angle is 30°.

| N. | | X | Y | Z | N. | | X | Y | Z |
|---|---|---|---|---|---|---|---|---|---|
| 1 | Co | 1.675 | 9.48 | 4.668 | 31 | N | -0.176 | 7.258 | 5.214 |
| 2 | N | 4.61885 | 9.94258 | 4.47986 | 32 | N | -1.222 | 8.842 | 4.238 |
| 3 | N | 3.52247 | 11.7771 | 4.56251 | 33 | C | 0.001 | 8.507 | 4.722 |
| 4 | C | 3.36 | 10.429 | 4.574 | 34 | C | -1.479 | 6.82 | 5.03 |
| 5 | C | 4.861 | 12.1209 | 4.47109 | 35 | C | -2.135 | 7.818 | 4.42 |
| 6 | C | 5.55012 | 10.9641 | 4.41981 | 36 | C | 0.89 | 6.49 | 5.81 |
| 7 | C | 4.90918 | 8.52382 | 4.40756 | 37 | C | 1.203 | 6.703 | 7.151 |
| 8 | C | 4.89657 | 7.91187 | 3.16084 | 38 | C | 2.228 | 5.935 | 7.698 |
| 9 | C | 5.16214 | 6.54801 | 3.11047 | 39 | C | 2.908 | 4.99 | 6.951 |
| 10 | C | 5.45885 | 5.81289 | 4.25103 | 40 | C | 2.585 | 4.83 | 5.62 |
| 11 | C | 5.46147 | 6.46552 | 5.46628 | 41 | C | 1.579 | 5.582 | 5.016 |
| 12 | C | 5.16969 | 7.82654 | 5.57775 | 42 | C | -1.52 | 10.106 | 3.606 |
| 13 | C | 2.40242 | 12.6697 | 4.66444 | 43 | C | -2.14 | 11.097 | 4.361 |
| 14 | C | 1.72825 | 13.0402 | 3.50621 | 44 | C | -2.49 | 12.277 | 3.701 |
| 15 | C | 0.587906 | 13.8293 | 3.65268 | 45 | C | -2.229 | 12.48 | 2.373 |
| 16 | C | 0.138958 | 14.236 | 4.88656 | 46 | C | -1.574 | 11.485 | 1.663 |
| 17 | C | 0.838891 | 13.853 | 6.01544 | 47 | C | -1.197 | 10.275 | 2.259 |
| 18 | C | 1.97478 | 13.0558 | 5.93482 | 48 | H | -1.836 | 5.978 | 5.287 |
| 19 | H | 5.22307 | 12.9994 | 4.44936 | 49 | H | -3.049 | 7.822 | 4.164 |
| 20 | H | 6.49457 | 10.8679 | 4.35431 | 50 | H | 0.760068 | 7.35889 | 7.67647 |
| 21 | H | 4.70726 | 8.40277 | 2.36985 | 51 | H | 2.467 | 6.062 | 8.61 |
| 22 | H | 5.1387 | 6.10546 | 2.26943 | 52 | H | 3.5967 | 4.47206 | 7.35091 |
| 23 | H | 5.65339 | 4.88541 | 4.18433 | 53 | H | 3.061 | 4.192 | 5.101 |
| 24 | H | 5.66919 | 5.9733 | 6.25221 | 54 | H | 1.40279 | 5.49737 | 4.08633 |
| 25 | H | 5.12841 | 8.2425 | 6.43085 | 55 | H | -2.32296 | 10.9714 | 5.28472 |
| 26 | H | 2.01563 | 12.743 | 2.6509 | 56 | H | -2.926 | 12.963 | 4.193 |
| 27 | H | 0.105564 | 14.0932 | 2.87881 | 57 | H | -2.49042 | 13.2887 | 1.94858 |
| 28 | H | -0.63727 | 14.7779 | 4.96608 | 58 | H | -1.376 | 11.629 | 0.745 |
| 29 | H | 0.536462 | 14.1418 | 6.86788 | 59 | H | -0.72989 | 9.60653 | 1.7717 |
| 30 | H | 2.4322 | 12.7646 | 6.71486 | | | | | |

**Table S5**. Atomic coordinates of model $[Co(IPh)_2]^{1+}$ in Cartesian coordinate system. The dihedral angle is 50°.

| N. | | X | Y | Z | N. | | X | Y | Z |
|---|---|---|---|---|---|---|---|---|---|
| 1 | Co | 1.675 | 9.48 | 4.668 | 31 | N | -0.176 | 7.258 | 5.214 |
| 2 | N | 4.56429 | 10.0045 | 4.12719 | 32 | N | -1.222 | 8.842 | 4.238 |
| 3 | N | 3.57526 | 11.7203 | 4.9349 | 33 | C | 0.001 | 8.507 | 4.722 |
| 4 | C | 3.36 | 10.429 | 4.574 | 34 | C | -1.479 | 6.82 | 5.03 |
| 5 | C | 4.89366 | 12.0878 | 4.72299 | 35 | C | -2.135 | 7.818 | 4.42 |
| 6 | C | 5.51527 | 11.0057 | 4.21485 | 36 | C | 0.89 | 6.49 | 5.81 |
| 7 | C | 4.77839 | 8.67502 | 3.58956 | 37 | C | 1.203 | 6.703 | 7.151 |
| 8 | C | 4.53423 | 8.46388 | 2.23873 | 38 | C | 2.228 | 5.935 | 7.698 |
| 9 | C | 4.72987 | 7.18 | 1.74219 | 39 | C | 2.908 | 4.99 | 6.951 |
| 10 | C | 5.17942 | 6.14013 | 2.54587 | 40 | C | 2.585 | 4.83 | 5.62 |
| 11 | C | 5.41026 | 6.39941 | 3.88088 | 41 | C | 1.579 | 5.582 | 5.016 |
| 12 | C | 5.19876 | 7.66208 | 4.43848 | 42 | C | -1.52 | 10.106 | 3.606 |
| 13 | C | 2.52621 | 12.5312 | 5.48571 | 43 | C | -2.14 | 11.097 | 4.361 |
| 14 | C | 1.68637 | 13.225 | 4.62165 | 44 | C | -2.49 | 12.277 | 3.701 |
| 15 | C | 0.620296 | 13.9238 | 5.18712 | 45 | C | -2.229 | 12.48 | 2.373 |
| 16 | C | 0.39884 | 13.9387 | 6.54366 | 46 | C | -1.574 | 11.485 | 1.663 |
| 17 | C | 1.25869 | 13.242 | 7.37203 | 47 | C | -1.197 | 10.275 | 2.259 |
| 18 | C | 2.3309 | 12.5158 | 6.86679 | 48 | H | -1.836 | 5.978 | 5.287 |
| 19 | H | 5.28404 | 12.9359 | 4.90119 | 49 | H | -3.049 | 7.822 | 4.164 |
| 20 | H | 6.43101 | 10.9421 | 3.96407 | 50 | H | 0.760068 | 7.35889 | 7.67647 |
| 21 | H | 4.23788 | 9.16714 | 1.67296 | 51 | H | 2.467 | 6.062 | 8.61 |
| 22 | H | 4.54919 | 7.00905 | 0.824649 | 52 | H | 3.5967 | 4.47206 | 7.35091 |
| 23 | H | 5.32084 | 5.27688 | 2.17534 | 53 | H | 3.061 | 4.192 | 5.101 |
| 24 | H | 5.72385 | 5.69658 | 4.43838 | 54 | H | 1.40279 | 5.49737 | 4.08633 |
| 25 | H | 5.31653 | 7.80344 | 5.37049 | 55 | H | -2.32296 | 10.9714 | 5.28472 |
| 26 | H | 1.81576 | 13.1999 | 3.68084 | 56 | H | -2.926 | 12.963 | 4.193 |
| 27 | H | 0.02842 | 14.4026 | 4.62006 | 57 | H | -2.49042 | 13.2887 | 1.94858 |
| 28 | H | -0.32996 | 14.4249 | 6.91101 | 58 | H | -1.376 | 11.629 | 0.745 |
| 29 | H | 1.11364 | 13.2599 | 8.31022 | 59 | H | -0.72989 | 9.60653 | 1.7717 |
| 30 | H | 2.89808 | 12.0088 | 7.4358 | | | | | |

**Table S6**. Atomic coordinates of model $[Co(IPh)_2]^{1+}$ in Cartesian coordinate system. The dihedral angle is 60°.

| N. | | X | Y | Z | N. | | X | Y | Z |
|---|---|---|---|---|---|---|---|---|---|
| 1 | Co | 1.675 | 9.48 | 4.668 | 31 | N | -0.176 | 7.258 | 5.214 |
| 2 | N | 4.51578 | 10.0747 | 3.96669 | 32 | N | -1.222 | 8.842 | 4.238 |
| 3 | N | 3.62421 | 11.6503 | 5.10564 | 33 | C | 0.001 | 8.507 | 4.722 |
| 4 | C | 3.36 | 10.429 | 4.574 | 34 | C | -1.479 | 6.82 | 5.03 |
| 5 | C | 4.92533 | 12.0431 | 4.8393 | 35 | C | -2.135 | 7.818 | 4.42 |
| 6 | C | 5.48559 | 11.0492 | 4.12241 | 36 | C | 0.89 | 6.49 | 5.81 |
| 7 | C | 4.66385 | 8.84163 | 3.21842 | 37 | C | 1.203 | 6.703 | 7.151 |
| 8 | C | 4.30334 | 8.83803 | 1.87716 | 38 | C | 2.228 | 5.935 | 7.698 |
| 9 | C | 4.43717 | 7.64381 | 1.17781 | 39 | C | 2.908 | 4.99 | 6.951 |
| 10 | C | 4.93744 | 6.49332 | 1.77399 | 40 | C | 2.585 | 4.83 | 5.62 |
| 11 | C | 5.28407 | 6.54692 | 3.10806 | 41 | C | 1.579 | 5.582 | 5.016 |
| 12 | C | 5.13928 | 7.71096 | 3.8657 | 42 | C | -1.52 | 10.106 | 3.606 |
| 13 | C | 2.63764 | 12.3705 | 5.86033 | 43 | C | -2.14 | 11.097 | 4.361 |
| 14 | C | 1.73826 | 13.1888 | 5.18599 | 44 | C | -2.49 | 12.277 | 3.701 |
| 15 | C | 0.734297 | 13.7959 | 5.93965 | 45 | C | -2.229 | 12.48 | 2.373 |
| 16 | C | 0.628468 | 13.6056 | 7.29687 | 46 | C | -1.574 | 11.485 | 1.663 |
| 17 | C | 1.54473 | 12.7897 | 7.93391 | 47 | C | -1.197 | 10.275 | 2.259 |
| 18 | C | 2.55948 | 12.1464 | 7.2349 | 48 | H | -1.836 | 5.978 | 5.287 |
| 19 | H | 5.34197 | 12.8537 | 5.10919 | 49 | H | -3.049 | 7.822 | 4.164 |
| 20 | H | 6.37582 | 11.0226 | 3.78731 | 50 | H | 0.760068 | 7.35889 | 7.67647 |
| 21 | H | 3.97078 | 9.61946 | 1.45141 | 51 | H | 2.467 | 6.062 | 8.61 |
| 22 | H | 4.17709 | 7.61418 | 0.263898 | 52 | H | 3.5967 | 4.47206 | 7.35091 |
| 23 | H | 5.03417 | 5.69589 | 1.2668 | 53 | H | 3.061 | 4.192 | 5.101 |
| 24 | H | 5.63312 | 5.76714 | 3.52425 | 54 | H | 1.40279 | 5.49737 | 4.08633 |
| 25 | H | 5.33745 | 7.70928 | 4.7948 | 55 | H | -2.32296 | 10.9714 | 5.28472 |
| 26 | H | 1.78732 | 13.3062 | 4.24455 | 56 | H | -2.926 | 12.963 | 4.193 |
| 27 | H | 0.103842 | 14.3562 | 5.50413 | 57 | H | -2.49042 | 13.2887 | 1.94858 |
| 28 | H | -0.05937 | 14.032 | 7.79445 | 58 | H | -1.376 | 11.629 | 0.745 |
| 29 | H | 1.47974 | 12.6656 | 8.87302 | 59 | H | -0.72989 | 9.60653 | 1.7717 |
| 30 | H | 3.16508 | 11.5579 | 7.6702 | | | | | |

**Table S7**. Atomic coordinates of model $[Co(IPh)_2]^{1+}$ in Cartesian coordinate system. The dihedral angle is 70°.

| N. | | X | Y | Z | N. | | X | Y | Z |
|---|---|---|---|---|---|---|---|---|---|
| 1 | Co | 1.675 | 9.48 | 4.668 | 31 | N | -0.176 | 7.258 | 5.214 |
| 2 | N | 4.45494 | 10.1686 | 3.82337 | 32 | N | -1.222 | 8.842 | 4.238 |
| 3 | N | 3.68635 | 11.5551 | 5.25905 | 33 | C | 0.001 | 8.507 | 4.722 |
| 4 | C | 3.36 | 10.429 | 4.574 | 34 | C | -1.479 | 6.82 | 5.03 |
| 5 | C | 4.96604 | 11.9812 | 4.94439 | 35 | C | -2.135 | 7.818 | 4.42 |
| 6 | C | 5.44885 | 11.1063 | 4.04048 | 36 | C | 0.89 | 6.49 | 5.81 |
| 7 | C | 4.52086 | 9.06276 | 2.88784 | 37 | C | 1.203 | 6.703 | 7.151 |
| 8 | C | 4.04833 | 9.26309 | 1.59728 | 38 | C | 2.228 | 5.935 | 7.698 |
| 9 | C | 4.10478 | 8.18833 | 0.71701 | 39 | C | 2.908 | 4.99 | 6.951 |
| 10 | C | 4.63642 | 6.95983 | 1.0878 | 40 | C | 2.585 | 4.83 | 5.62 |
| 11 | C | 5.09526 | 6.81005 | 2.38001 | 41 | C | 1.579 | 5.582 | 5.016 |
| 12 | C | 5.03236 | 7.84616 | 3.31408 | 42 | C | -1.52 | 10.106 | 3.606 |
| 13 | C | 2.77792 | 12.1546 | 6.19549 | 43 | C | -2.14 | 11.097 | 4.361 |
| 14 | C | 1.83708 | 13.0674 | 5.73186 | 44 | C | -2.49 | 12.277 | 3.701 |
| 15 | C | 0.909538 | 13.5553 | 6.65172 | 45 | C | -2.229 | 12.48 | 2.373 |
| 16 | C | 0.915909 | 13.1618 | 7.96876 | 46 | C | -1.574 | 11.485 | 1.663 |
| 17 | C | 1.87044 | 12.257 | 8.39457 | 47 | C | -1.197 | 10.275 | 2.259 |
| 18 | C | 2.81281 | 11.725 | 7.52213 | 48 | H | -1.836 | 5.978 | 5.287 |
| 19 | H | 5.41603 | 12.7407 | 5.29666 | 49 | H | -3.049 | 7.822 | 4.164 |
| 20 | H | 6.30711 | 11.129 | 3.6301 | 50 | H | 0.760068 | 7.35889 | 7.67647 |
| 21 | H | 3.69268 | 10.1006 | 1.32431 | 51 | H | 2.467 | 6.062 | 8.61 |
| 22 | H | 3.76803 | 8.29799 | -0.16522 | 52 | H | 3.5967 | 4.47206 | 7.35091 |
| 23 | H | 4.67806 | 6.24825 | 0.459781 | 53 | H | 3.061 | 4.192 | 5.101 |
| 24 | H | 5.46655 | 5.97554 | 2.6424 | 54 | H | 1.40279 | 5.49737 | 4.08633 |
| 25 | H | 5.30826 | 7.70337 | 4.21184 | 55 | H | -2.32296 | 10.9714 | 5.28472 |
| 26 | H | 1.80819 | 13.326 | 4.81818 | 56 | H | -2.926 | 12.963 | 4.193 |
| 27 | H | 0.252973 | 14.1764 | 6.36135 | 57 | H | -2.49042 | 13.2887 | 1.94858 |
| 28 | H | 0.278998 | 13.5092 | 8.58206 | 58 | H | -1.376 | 11.629 | 0.745 |
| 29 | H | 1.88317 | 11.9922 | 9.3063 | 59 | H | -0.72989 | 9.60653 | 1.7717 |
| 30 | H | 3.44418 | 11.0762 | 7.81019 | | | | | |

**Table S8**. Atomic coordinates of model $[Co(IPh)_2]^{1+}$ in Cartesian coordinate system. The dihedral angle is 80°.

| N. | | X | Y | Z | N. | | X | Y | Z |
|---|---|---|---|---|---|---|---|---|---|
| 1 | Co | 1.675 | 9.48 | 4.668 | 31 | N | -0.176 | 7.258 | 5.214 |
| 2 | N | 4.38362 | 10.2832 | 3.70159 | 32 | N | -1.222 | 8.842 | 4.238 |
| 3 | N | 3.75981 | 11.4377 | 5.39046 | 33 | C | 0.001 | 8.507 | 4.722 |
| 4 | C | 3.36 | 10.429 | 4.574 | 34 | C | -1.479 | 6.82 | 5.03 |
| 5 | C | 5.01456 | 11.9041 | 5.03507 | 35 | C | -2.135 | 7.818 | 4.42 |
| 6 | C | 5.40619 | 11.1752 | 3.97155 | 36 | C | 0.89 | 6.49 | 5.81 |
| 7 | C | 4.35378 | 9.33169 | 2.60786 | 37 | C | 1.203 | 6.703 | 7.151 |
| 8 | C | 3.77696 | 9.72613 | 1.40759 | 38 | C | 2.228 | 5.935 | 7.698 |
| 9 | C | 3.74282 | 8.79702 | 0.37379 | 39 | C | 2.908 | 4.99 | 6.951 |
| 10 | C | 4.2855 | 7.52549 | 0.50816 | 40 | C | 2.585 | 4.83 | 5.62 |
| 11 | C | 4.84957 | 7.18079 | 1.71885 | 41 | C | 1.579 | 5.582 | 5.016 |
| 12 | C | 4.88125 | 8.06357 | 2.80036 | 42 | C | -1.52 | 10.106 | 3.606 |
| 13 | C | 2.94276 | 11.8902 | 6.48099 | 43 | C | -2.14 | 11.097 | 4.361 |
| 14 | C | 1.97985 | 12.8645 | 6.24269 | 44 | C | -2.49 | 12.277 | 3.701 |
| 15 | C | 1.14069 | 13.2092 | 7.30169 | 45 | C | -2.229 | 12.48 | 2.373 |
| 16 | C | 1.25243 | 12.6208 | 8.5389 | 46 | C | -1.574 | 11.485 | 1.663 |
| 17 | C | 2.22592 | 11.6601 | 8.74 | 47 | C | -1.197 | 10.275 | 2.259 |
| 18 | C | 3.08319 | 11.2645 | 7.71976 | 48 | H | -1.836 | 5.978 | 5.287 |
| 19 | H | 5.50396 | 12.6006 | 5.45791 | 49 | H | -3.049 | 7.822 | 4.164 |
| 20 | H | 6.22695 | 11.2582 | 3.49721 | 50 | H | 0.760068 | 7.35889 | 7.67647 |
| 21 | H | 3.41205 | 10.5961 | 1.29553 | 51 | H | 2.467 | 6.062 | 8.61 |
| 22 | H | 3.33442 | 9.03972 | -0.44965 | 52 | H | 3.5967 | 4.47206 | 7.35091 |
| 23 | H | 4.26333 | 6.91718 | -0.22119 | 53 | H | 3.061 | 4.192 | 5.101 |
| 24 | H | 5.22921 | 6.31544 | 1.81961 | 54 | H | 1.40279 | 5.49737 | 4.08633 |
| 25 | H | 5.22985 | 7.78589 | 3.63934 | 55 | H | -2.32296 | 10.9714 | 5.28472 |
| 26 | H | 1.87773 | 13.2586 | 5.3843 | 56 | H | -2.926 | 12.963 | 4.193 |
| 27 | H | 0.471283 | 13.8684 | 7.16566 | 57 | H | -2.49042 | 13.2887 | 1.94858 |
| 28 | H | 0.674851 | 12.8725 | 9.2499 | 58 | H | -1.376 | 11.629 | 0.745 |
| 29 | H | 2.31166 | 11.2602 | 9.5969 | 59 | H | -0.72989 | 9.60653 | 1.7717 |
| 30 | H | 3.72688 | 10.5784 | 7.8515 | | | | | |

**Table S9**. Atomic coordinates of model $[Co(IPh)_2]^{1+}$ in Cartesian coordinate system. The dihedral angle is 90°.

| N. | | X | Y | Z | N. | | X | Y | Z |
|---|---|---|---|---|---|---|---|---|---|
| 1 | Co | 1.675 | 9.48 | 4.668 | 31 | N | -0.176 | 7.258 | 5.214 |
| 2 | N | 4.30397 | 10.415 | 3.60505 | 32 | N | -1.222 | 8.842 | 4.238 |
| 3 | N | 3.84235 | 11.3016 | 5.49588 | 33 | C | 0.001 | 8.507 | 4.722 |
| 4 | C | 3.36 | 10.429 | 4.574 | 34 | C | -1.479 | 6.82 | 5.03 |
| 5 | C | 5.0694 | 11.814 | 5.10858 | 35 | C | -2.135 | 7.818 | 4.42 |
| 6 | C | 5.35888 | 11.2539 | 3.91771 | 36 | C | 0.89 | 6.49 | 5.81 |
| 7 | C | 4.16767 | 9.64026 | 2.38701 | 37 | C | 1.203 | 6.703 | 7.151 |
| 8 | C | 3.49747 | 10.2131 | 1.31386 | 38 | C | 2.228 | 5.935 | 7.698 |
| 9 | C | 3.36228 | 9.45138 | 0.15858 | 39 | C | 2.908 | 4.99 | 6.951 |
| 10 | C | 3.89535 | 8.17312 | 0.052677 | 40 | C | 2.585 | 4.83 | 5.62 |
| 11 | C | 4.55447 | 7.64789 | 1.14468 | 41 | C | 1.579 | 5.582 | 5.016 |
| 12 | C | 4.69055 | 8.35659 | 2.34017 | 42 | C | -1.52 | 10.106 | 3.606 |
| 13 | C | 3.12717 | 11.5853 | 6.70816 | 43 | C | -2.14 | 11.097 | 4.361 |
| 14 | C | 2.16221 | 12.5863 | 6.70294 | 44 | C | -2.49 | 12.277 | 3.701 |
| 15 | C | 1.42074 | 12.7682 | 7.86981 | 45 | C | -2.229 | 12.48 | 2.373 |
| 16 | C | 1.6278 | 11.9989 | 8.98998 | 46 | C | -1.574 | 11.485 | 1.663 |
| 17 | C | 2.60037 | 11.017 | 8.95971 | 47 | C | -1.197 | 10.275 | 2.259 |
| 18 | C | 3.36239 | 10.7789 | 7.82179 | 48 | H | -1.836 | 5.978 | 5.287 |
| 19 | H | 5.60311 | 12.4374 | 5.58805 | 49 | H | -3.049 | 7.822 | 4.164 |
| 20 | H | 6.13779 | 11.4061 | 3.39268 | 50 | H | 0.760068 | 7.35889 | 7.67647 |
| 21 | H | 3.13742 | 11.0907 | 1.36594 | 51 | H | 2.467 | 6.062 | 8.61 |
| 22 | H | 2.88944 | 9.81681 | -0.58077 | 52 | H | 3.5967 | 4.47206 | 7.35091 |
| 23 | H | 3.80257 | 7.68235 | -0.75543 | 53 | H | 3.061 | 4.192 | 5.101 |
| 24 | H | 4.92832 | 6.77653 | 1.08089 | 54 | H | 1.40279 | 5.49737 | 4.08633 |
| 25 | H | 5.10459 | 7.95434 | 3.09467 | 55 | H | -2.32296 | 10.9714 | 5.28472 |
| 26 | H | 1.99384 | 13.106 | 5.92572 | 56 | H | -2.926 | 12.963 | 4.193 |
| 27 | H | 0.752139 | 13.4418 | 7.89264 | 57 | H | -2.49042 | 13.2887 | 1.94858 |
| 28 | H | 1.11616 | 12.1412 | 9.77768 | 58 | H | -1.376 | 11.629 | 0.745 |
| 29 | H | 2.75221 | 10.4917 | 9.73598 | 59 | H | -0.72989 | 9.60653 | 1.7717 |
| 30 | H | 4.00461 | 10.0794 | 7.79289 | | | | | |

**Table S10**. Atomic coordinates of model $[Co(sIPh')_2]^{1+}$ in Cartesian coordinate system.

| N. | | X | Y | Z | N. | | X | Y | Z |
|---|---|---|---|---|---|---|---|---|---|
| 1 | Co | 1.701 | 9.453 | 4.594 | 31 | C | 4.662 | 8.091 | 2.642 |
| 2 | N | -1.189 | 8.922 | 4.264 | 32 | C | 4.92 | 6.762 | 2.332 |
| 3 | N | -0.1 | 7.231 | 5.058 | 33 | C | 5.352 | 5.855 | 3.292 |
| 4 | N | 3.519 | 11.715 | 4.698 | 34 | C | 5.512 | 6.304 | 4.596 |
| 5 | N | 4.602 | 9.879 | 4.308 | 35 | C | 5.248 | 7.622 | 4.956 |
| 6 | C | 0.014 | 8.502 | 4.648 | 36 | H | -3.12758 | 7.9777 | 4.02662 |
| 7 | C | 3.391 | 10.392 | 4.522 | 37 | H | -1.76144 | 5.81146 | 5.11742 |
| 8 | C | -2.226 | 7.866 | 4.301 | 38 | H | -2.36332 | 11.0392 | 5.25661 |
| 9 | C | -1.486 | 6.709 | 4.979 | 39 | H | -3.015 | 12.981 | 4.129 |
| 10 | C | -1.462 | 10.179 | 3.624 | 40 | H | -2.49403 | 13.3142 | 1.8902 |
| 11 | C | -2.16 | 11.156 | 4.336 | 41 | H | -1.249 | 11.694 | 0.755 |
| 12 | C | -2.531 | 12.311 | 3.661 | 42 | H | -0.58395 | 9.72594 | 1.81185 |
| 13 | C | -2.221 | 12.521 | 2.336 | 43 | H | 0.888454 | 7.39102 | 7.49729 |
| 14 | C | -1.486 | 11.549 | 1.664 | 44 | H | 2.552 | 6.071 | 8.454 |
| 15 | C | -1.091 | 10.373 | 2.288 | 45 | H | 3.63045 | 4.40532 | 7.22273 |
| 16 | C | 0.971 | 6.48 | 5.652 | 46 | H | 3.065 | 4.1 | 4.982 |
| 17 | C | 1.305 | 6.711 | 6.981 | 47 | H | 1.45306 | 5.42573 | 3.95269 |
| 18 | C | 2.307 | 5.928 | 7.547 | 48 | H | 5.27379 | 13.0526 | 4.7755 |
| 19 | C | 2.961 | 4.939 | 6.811 | 49 | H | 6.61412 | 10.7958 | 4.23598 |
| 20 | C | 2.615 | 4.765 | 5.491 | 50 | H | 2.50511 | 12.2739 | 7.05138 |
| 21 | C | 1.634 | 5.525 | 4.88 | 51 | H | 0.65 | 13.617 | 7.538 |
| 22 | C | 4.91 | 12.184 | 4.658 | 52 | H | -0.57023 | 14.6453 | 5.8414 |
| 23 | C | 5.672 | 10.905 | 4.269 | 53 | H | 0.051 | 14.312 | 3.625 |
| 24 | C | 2.396 | 12.549 | 5.01 | 54 | H | 1.91774 | 13.0012 | 3.06528 |
| 25 | C | 2.029 | 12.706 | 6.352 | 55 | H | 4.38966 | 8.69318 | 1.95957 |
| 26 | C | 0.914 | 13.495 | 6.633 | 56 | H | 4.796 | 6.465 | 1.44 |
| 27 | C | 0.181 | 14.104 | 5.629 | 57 | H | 5.53323 | 4.96318 | 3.05659 |
| 28 | C | 0.561 | 13.909 | 4.315 | 58 | H | 5.812 | 5.695 | 5.261 |
| 29 | C | 1.672 | 13.136 | 3.973 | 59 | H | 5.33434 | 7.89362 | 5.86224 |
| 30 | C | 4.842 | 8.506 | 3.968 | | | | | |

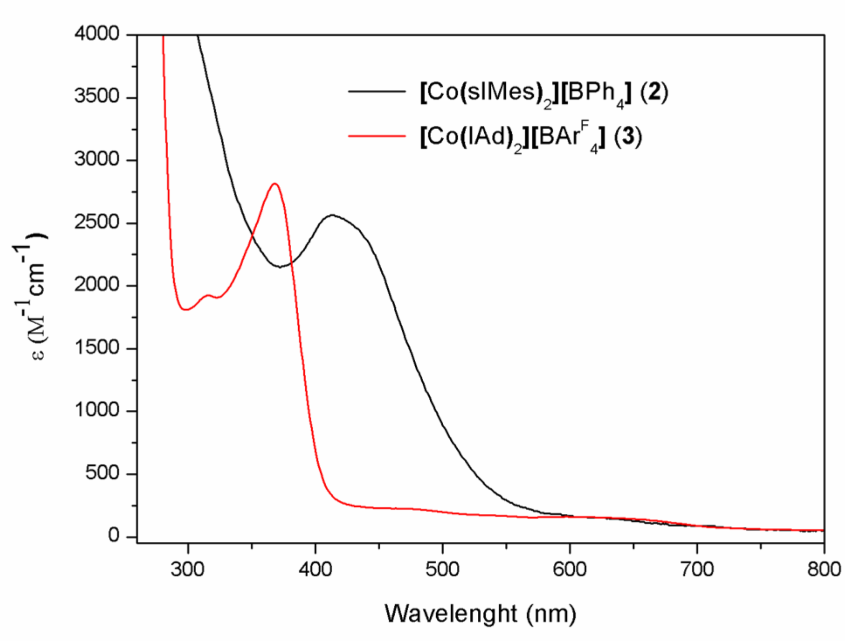


**Figure S1.** UV-vis Spectra of **2** and **3** Recorded at Room Temperature in THF.

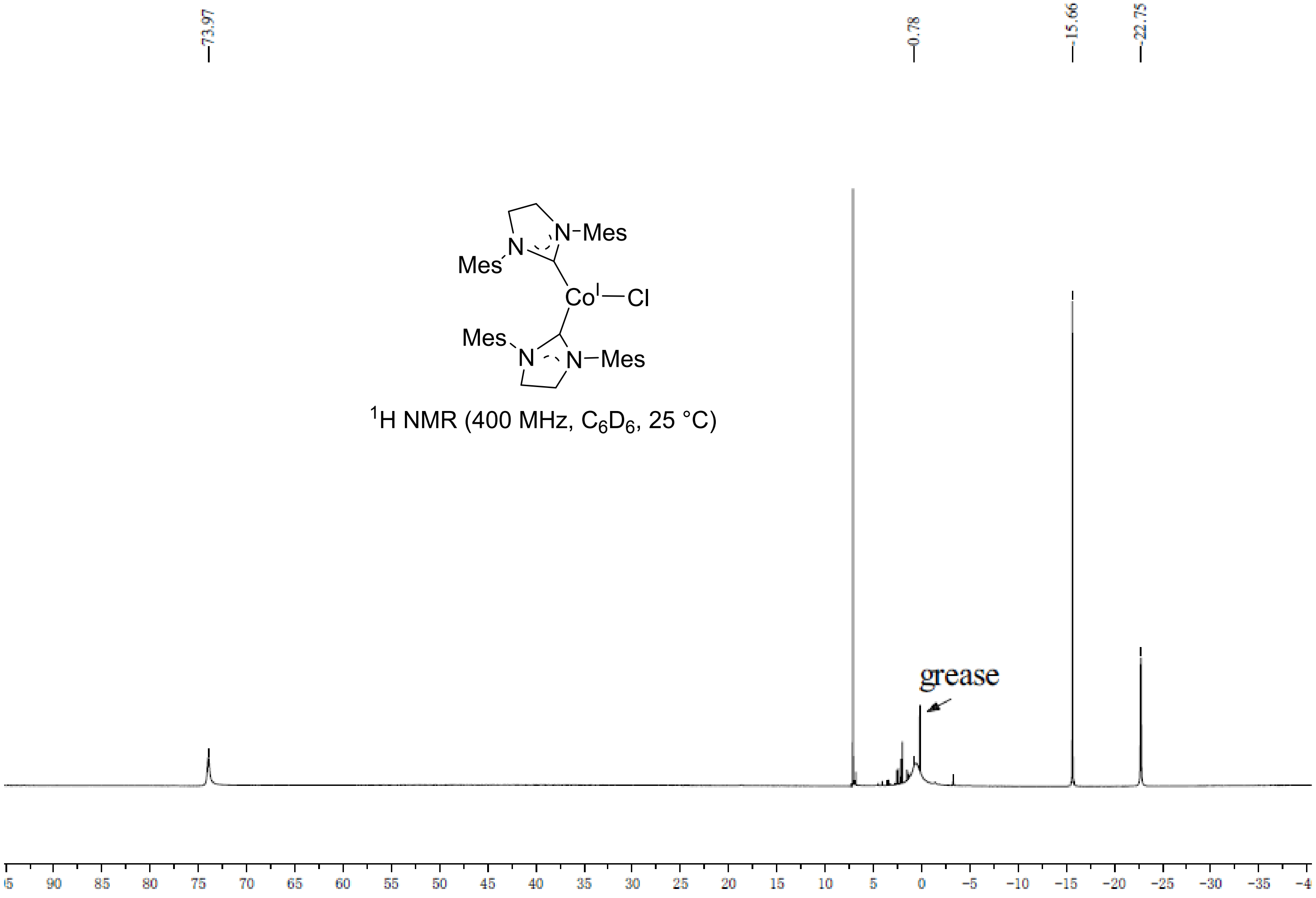


**Figure S2.** $^1$H NMR Spectrum of [Co(sIMes)$_2$Cl]

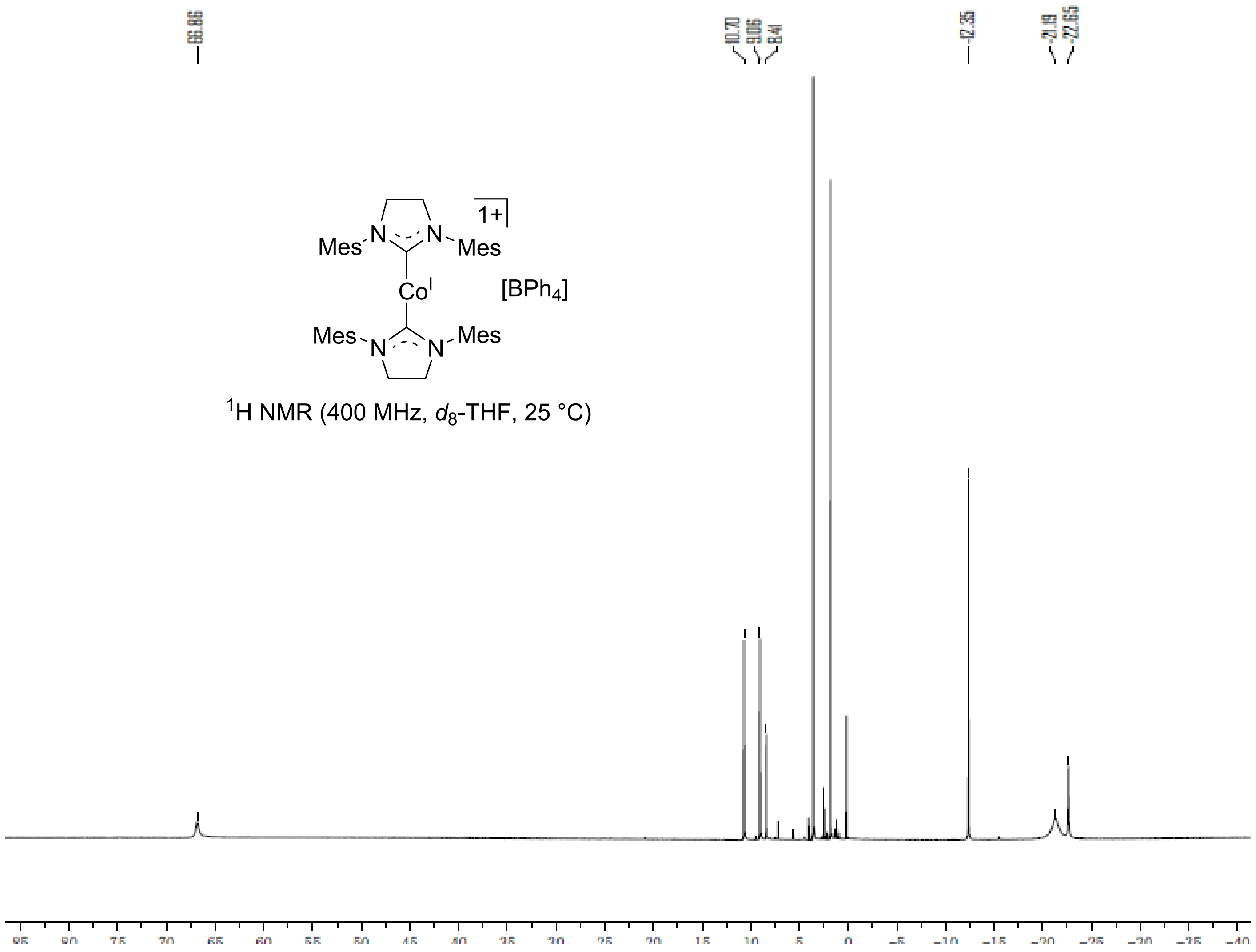


**Figure S3.** $^{1}$H NMR Spectrum of $[Co(sIMes)_2][BPh_4]$ (**2**)

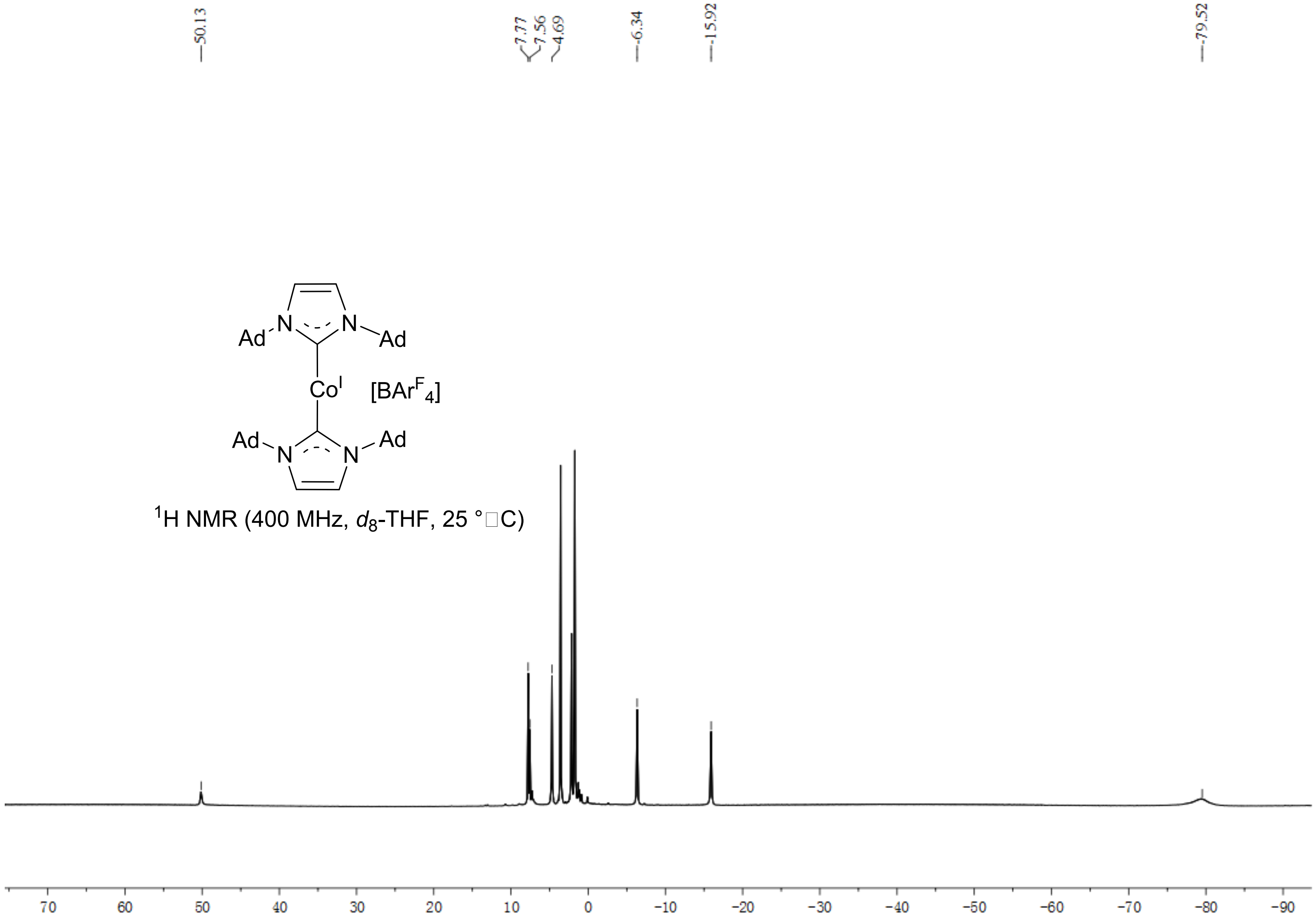


**Figure S4.** $^1$H NMR Spectrum of $[Co(IAd)_2][BAr^F_4]$ (**3**)

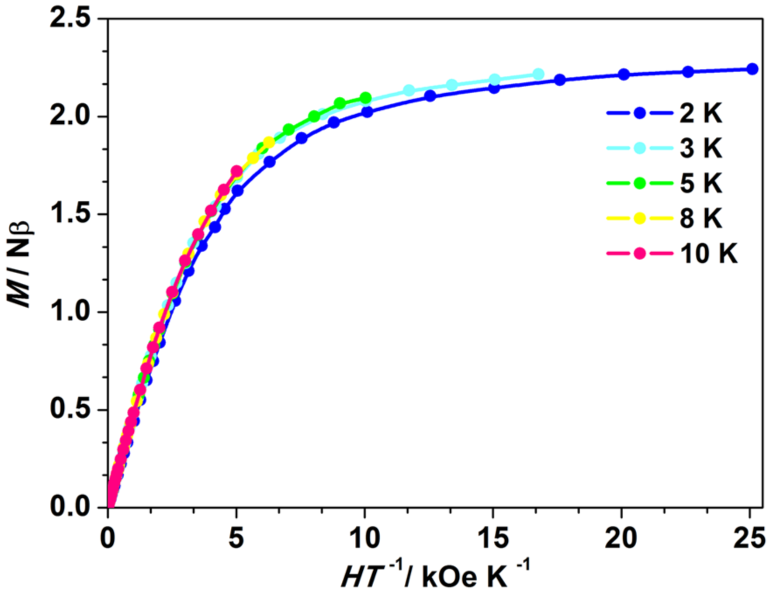


**Figure S5.** Variable-field isothermal susceptibility data of **1**.

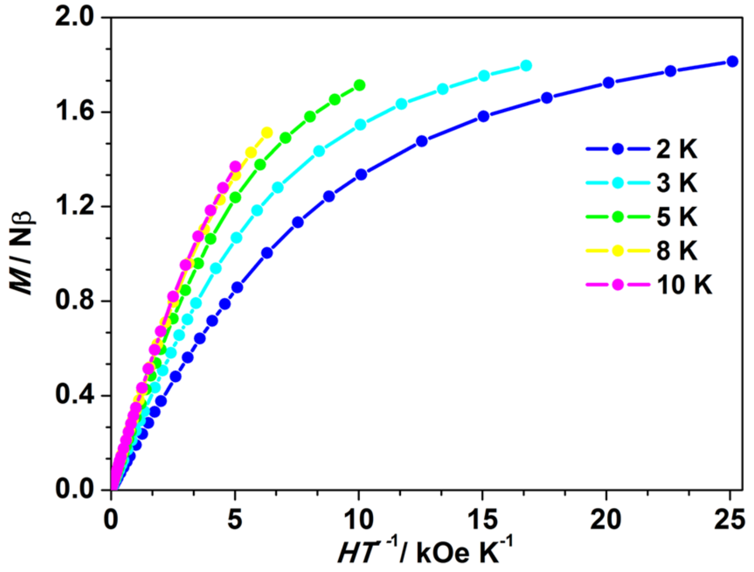


**Figure S6.** Variable-field isothermal susceptibility data of **2**.

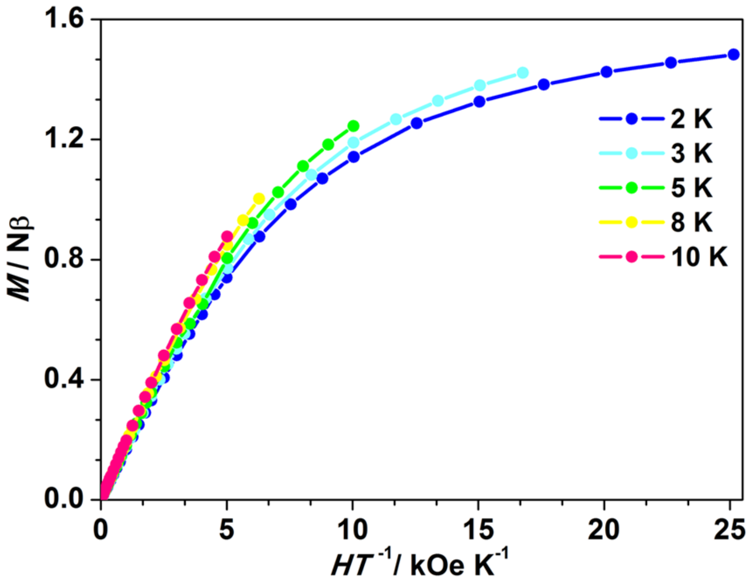


**Figure S7.** Variable-field isothermal susceptibility data of **3**.

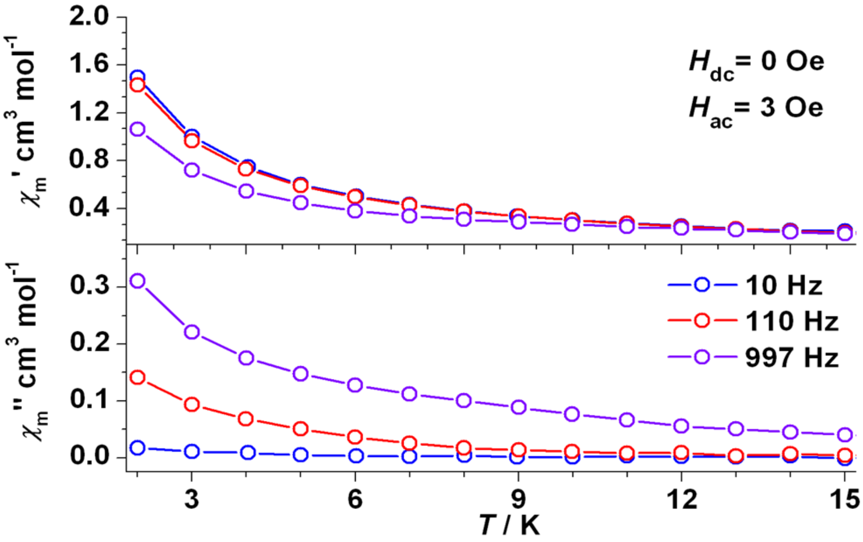


**Figure S8.** Temperature-dependent ac susceptibility data for **1** under zero dc field.

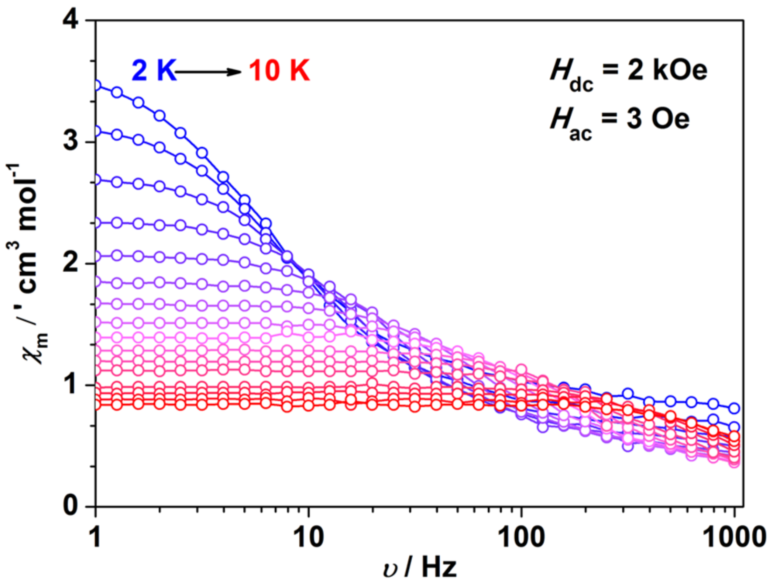


**Figure S9.** Frequency-dependent in-phase ac susceptibility data for **1** under 2 kOe dc field ranging from 2 to 10 K.

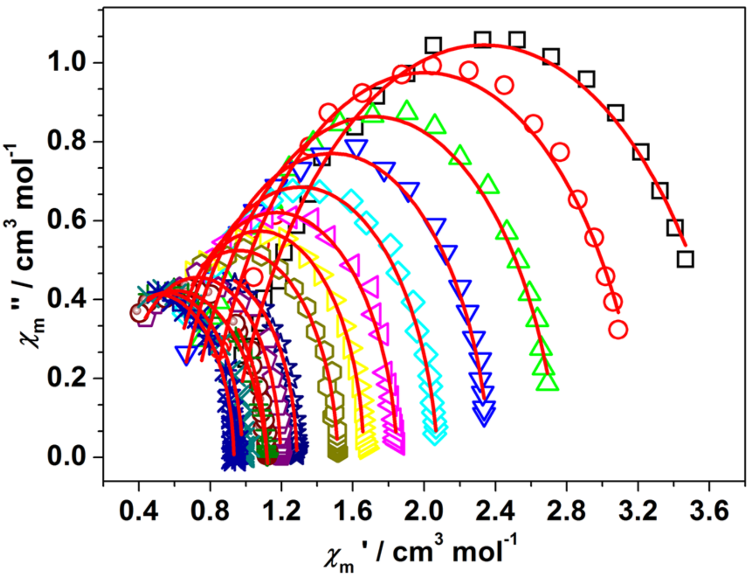


**Figure S10.** Cole-Cole plots fit for the determination of the temperature dependence of $\tau$ for **1** under 2 kOe dc field from 2 K to 9 K. Solid red lines represent the results of fitting to a generalized Debye model.

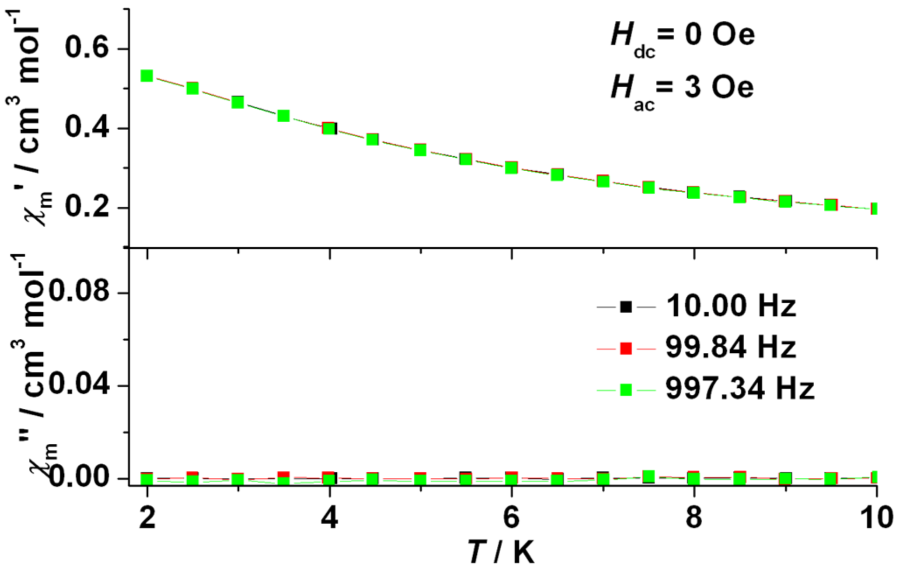


**Figure S11.** Temperature-dependent ac susceptibility data for **2** under zero dc field.

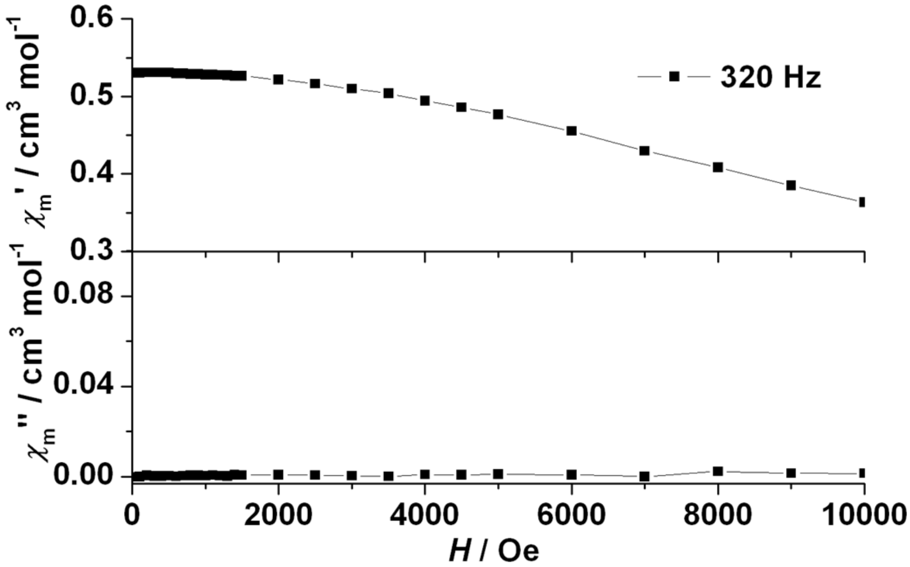


**Figure S12.** Ac susceptibility measurement by scanning the dc field at 2 K for **2**.

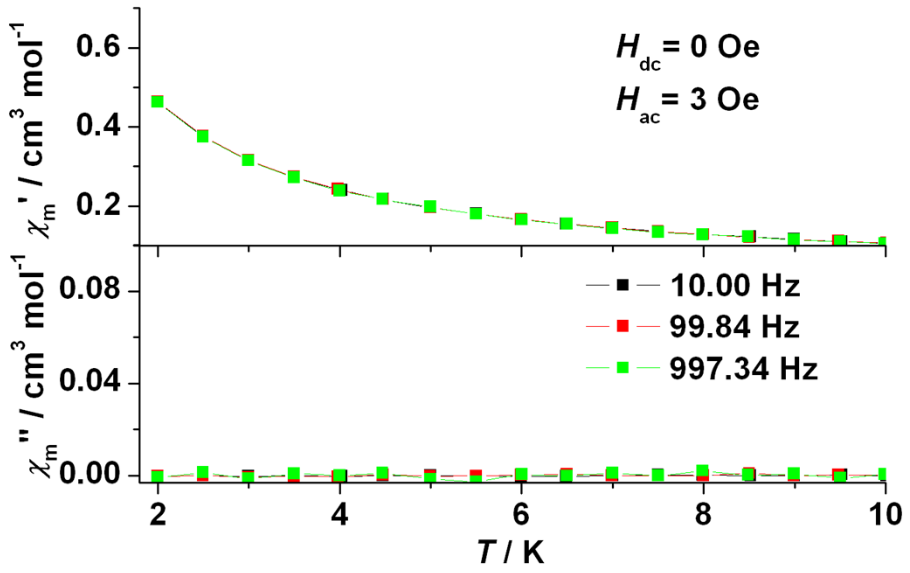


**Figure S13.** Temperature-dependent ac susceptibility data for **3** under zero dc field.

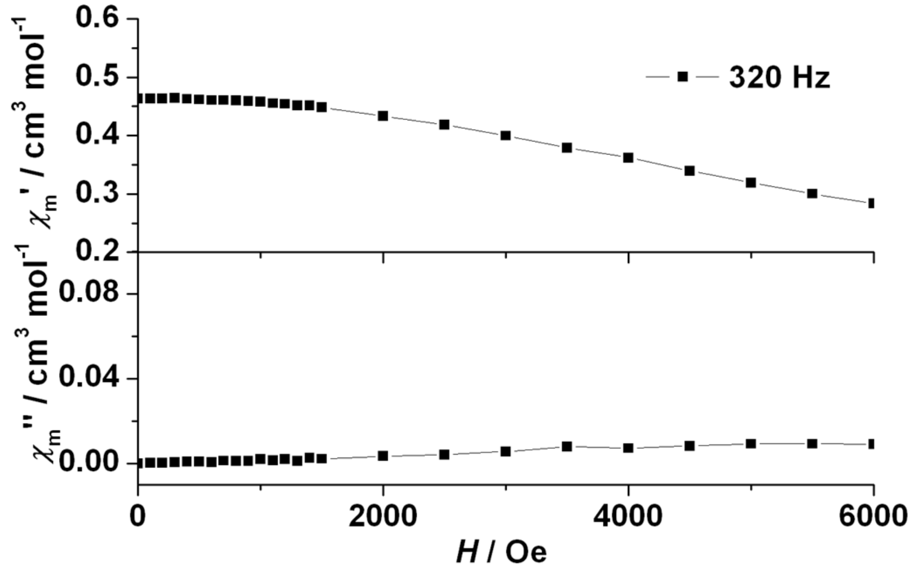


**Figure S14.** Ac susceptibility measurement by scanning the dc field at 2 K for **3**.
